# Temporal resolution of spike coding in feedforward networks with signal convergence and divergence

**DOI:** 10.1101/2024.07.08.602598

**Published:** 2024-07-11

**Authors:** Zach Mobille, Usama Bin Sikandar, Simon Sponberg, Hannah Choi

**Affiliations:** 1 School of Mathematics, Georgia Institute of Technology, Atlanta, GA 30332; 2 Quantitative Biosciences Program, Georgia Institute of Technology, Atlanta, GA 30332; 3 School of Physics, Georgia Institute of Technology, Atlanta, GA 30332; 4 School of Biological Sciences, Georgia Institute of Technology, Atlanta, GA 30332

## Abstract

Convergent and divergent structures in the networks that make up biological brains are found universally across many species and brain regions at various scales. Neurons in these networks fire action potentials, or “spikes”, whose precise timing is becoming increasingly appreciated as large sources of information about both sensory input and motor output. While previous theories on coding in convergent and divergent networks have largely neglected the role of precise spike timing, our model and analyses place this aspect at the forefront. For a suite of stimuli with different timescales, we demonstrate that structural bottlenecks (small groups of neurons) post-synaptic to network convergence have a stronger preference for spike timing codes than expansion layers created by structural divergence. Additionally, we found that a simple network model with similar convergence and divergence ratios to those found experimentally can reproduce the relative contribution of spike timing information about motor output in the hawkmoth *Manduca sexta*. Our simulations and analyses suggest a relationship between the level of convergent/divergent structure present in a feedforward network and the loss of stimulus information encoded by its population spike trains as their temporal resolution decreases, which could be confirmed experimentally across diverse neural systems in future studies. We further show that this relationship can be generalized across different models and measures, implying a potentially fundamental link between network structure and coding strategy using spikes.

## 1 Introduction

The neural systems of animals comprise networks with highly non-random topological structure [1–6]. The relationship between computation and connectivity in neural networks is multi-faceted and depends on the question being asked [7–9], but often it can be fruitful to focus on connectivity patterns, or “motifs,” that are observed more often in biological systems than would be expected in a totally random model network [10–13]. One particular structural motif that is common in many areas of the nervous system involves populations of neurons synapsing with other populations of a much different size. When a large population of neurons synapses with a much smaller population, it may be called a “convergent” pathway. If a small population synapses with a much larger population, we call this structure “divergent.” Examples where both structural convergence and divergence are present include the mammalian early visual system [14–17], mammalian cerebellum-like structures [18, 19] and the insect olfactory system [20]. A notable example is the divergence from 200 million mossy fibers to 50 billion granule cells and then convergence to 15 million Purkinje cells in the human cerebellum, a largely feedforward network. [21, 22].

Despite their ubiquity, convergent/divergent structures are only beginning to be understood from a functional point of view. Previous work has shown that network convergence synergizes with nonlinear activation functions to boost information coding [14]. Other studies have focused explicitly on bottlenecks (a small group of neurons post-synaptic to a much larger group of pre-synaptic neurons), demonstrating that modular structure increases their information transfer [23], and that they increase dimension while reducing noise in the expansion layer post-synaptic to them [18]. The models used in all of these studies were non-spiking, neglecting the biologically-relevant role of precisely-timed action potentials.

A related but distinct concept to numerical/structural bottlenecks in biological neural networks is that of the information bottleneck, which is a variational method for extracting the most relevant information that a random variable X has about another random variable Y by finding an optimal compressed representation $\tilde{X}$ [24]. This method optimizes the tradeoff between prediction and compression and has been used to shed light on learning [25] and optimal architectures [26] in deep neural networks. While the vanilla information bottleneck method is agnostic to any particular mapping between $\tilde{X}$ and Y, recent work has extended the idea by finding an $\tilde{X}$ that is specific to the decoder being used for downstream prediction [27], thus improving generalization in artificial neural networks. A similar variant of the information bottleneck was applied to neural data from the cells of the retina, showing that predictive information about future visual inputs can be encoded and compressed by neurons post-synaptic to the retina [28]. Despite these interesting and thought-provoking studies, it is still unclear how the structural bottlenecks present in biological neural networks affect coding with population spikes, whose precise timing can be a rich source of information [29–32].

The demand for understanding the implications of convergent and divergent structure for the information coding of a spiking neural network is especially high in light of growing experimental evidence showing that spike timing can encode significantly more information about inputs [33] and outputs [34] than spike count. At the sensory input level, millisecond-level variations in spike timings encode significant proprioceptive [35], visual [36], auditory [37], olfactory [38] and tactile [39] information. Similarly, at the output level, millisecond-precise spike timings have also been shown to encode a significant (and sometimes greater than spike count) amount of information across human movement [40], muscle coordination [34], songbird acoustic structures [41] and respiration [42], insect flight control, turning maneuvers [34, 43], and escape behaviors [44]. Thus, in the sensory and motor peripheries of the nervous system, the importance of precise spike timing has been well established.

The role of spike timing is not as well understood in the intermediate stages of processing between sensory and motor populations, which, in the context of vertebrate visuomotor pathways, involve several cascades of structural convergence and divergence from the early visual system to cortex [16, 17] and eventually through the cerebellum [19, 21, 22] to the spinal cord and commanding muscles. A classic modeling study suggests that the cortex, a large population of neurons post-synaptic to structural divergence, is more likely to use a population spike count code due to high variability in inter-spike intervals [45]. Other work argues on the basis of energy expenditure that rate/count coding can only explain around 15% of the activity in primary visual cortex [46], suggesting that other coding strategies may explain the rest [47]. From a purely quantitative perspective, single-neuron count codes are slow and information-poor, but robust to noise [48]. The activity of large populations of neurons following a structural divergence comprises a high-dimensional space and may therefore benefit from a collective count code due to the reduction in noise. On the other hand, spike timing codes are fast, information-rich, but potentially sensitive to noise [49]. It is therefore possible that bottleneck populations of neurons post-synaptic to structural convergences may be good candidates for a temporal code, since this would allow them to encode a similar amount of information as the larger pre-synaptic layer but with a smaller number of neurons. Indeed, experiments testing white noise optogenetic stimuli in the cortex of mice have shown that temporal precision of spiking increases in the inter-neurons post-synaptic to a structural convergence compared to the pyramidal neurons pre-synaptic to them [50]. This is just one example, however, and it would improve our understanding of the information processing between sensory input and motor output if a generalizable relationship between population spike coding and convergent/divergent structure were established.

Thus, our aim is to study this relationship systematically in spiking neural network models. The primary hypothesis we advance here is that temporal coding is more beneficial in bottleneck populations post-synaptic to a structural convergence than it is in an expansion layer. While expansion layers have a surplus of neurons and may represent stimuli just fine with a coarse count code, bottlenecks have fewer neurons that can be employed to encode signals and may preserve information by expressing their representations in time. To test this hypothesis, we train feedforward spiking neural networks [47] and performing decoding analyses [51] on the population spike trains binned at various resolutions. First, we study 3-layered feedforward networks with varying levels of convergence and divergence to establish a general relationship between structure and spike coding. Next, we developed a 5-layered model resembling the patterns of expansion and contraction in a hawkmoth visuomotor pathway, whose output is known to use a spike timing code during hover-feeding and target tracking [34], and test our model predictions. To confirm that our results are not an exception due to a specific spiking model and decoder we chose, we also tested the robustness of the results using other models and measures.

## 2 Results

**Figure 1 ∣:**
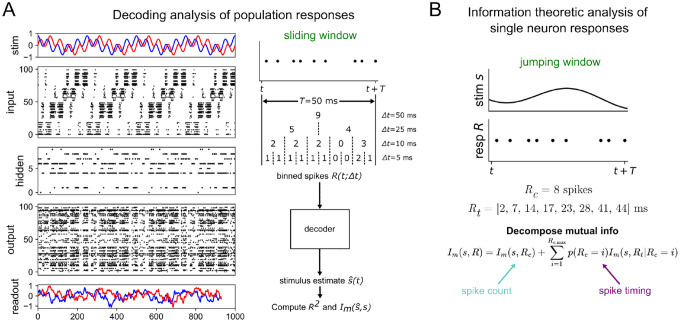
Schematic of model and analysis methods (A) Raster plot of 3-layer network model trained to a 4 Hz + 20 Hz sum of sines stimulus (left) and a depiction of the procedure used to process population spike trains before feeding them to the decoder to estimate the stimulus (right). T=50ms is the width of the sliding window used here and Δt is the bin size (B) Sketch of the information theoretic method used to validate the 5-layer network model against previous results from hawkmoth data. A jumping window of duration T=50 ms is used in this analysis.

A graphical summary of our approach is shown in Fig. 1. We first train a feedforward spiking neural network with a given structure to autoencode a time-dependent stimulus s(t) (left of Fig. 1A). To test how the encoding changes as we increase the temporal resolution of the spike trains we use a decoding analysis in which we process each layer’s spikes over a sliding window of width T=50ms that are further binned at resolution Δt. These binned spikes R(t;Δt) are then fed to a decoder (a recurrent neural network) that treats the binned spikes within the larger response window as a sequence of hidden states within its own dynamics. The job of the decoder is to estimate the stimulus presented to the input layer of the network with a reconstruction $\overset{ˆ}{s}$, based on the binned population spiking of the layer of interest. We then quantify the relationship between response R(t;Δt) and stimulus s(t) by computing both the decoding accuracy $R^{2}$ and the mutual information $I_{m}(s,\overset{ˆ}{s})$ for various Δt. These measures approximate the true information carried at the population level and are computed across a range of Δt to establish the temporal resolution of the optimal coding strategy, referred to here as the “information curves”.

We also perform an information theoretic analysis at the single-neuron level, based on past work [34, 52]. The strength of this method is that it separates spike count and spike timing information in a clean way. Note that the binning method used in the population analysis considers spike counts over increasing levels of time resolution and therefore does not isolate spike-timing code completely from spike-count code, while having its strength in considering all the neurons in the layer simultaneously. The mutual information at the single-cell resolution also allows us to compare our model’s results with previously obtained experimental results at the single neuron level. For a detailed explanation of our model and analysis, see Section 4.

### 2.1 Three-layer network

We first focus on a feedforward network of 3 layers, systematically varying the number of neurons in the middle layer while keeping the number of neurons in the input and output layer fixed at $N_{\text{in}}=N_{\text{out}}=100$. By doing this, we simultaneously tune the level of structural divergence and convergence. The network model consists of leaky integrate-and-fire neurons with both excitatory and inhibitory synapses. The parameters of the network, including synaptic weights and membrane time constants, are optimized to minimize the following loss function

(1) $${ℒ}_{\text{MSE}}(z,s)=\frac{1}{N_{t}}\sum_{t=1}^{N_{t}}\;{\left(z_{t}-s_{t}\right)}^{2}$$

where $N_{t}$ is the total number of time points, $s_{t}$ is the true stimulus at time t, and z is a readout from the output layer of the form

(2) $$z=\gamma z_{\text{time}}+(1-\gamma )z_{\text{count}}$$

where $z_{\text{time}}$ is a readout based on the spike timings of the output layer and $z_{\text{count}}$ is a readout based on the spike counts of the output layer. The quantity γ is a hyperparameter that we set to 0.5, so as to equally weigh the readouts based on spike count and spike timing, thus not biasing our results (for more details, see Methods).

After training the network, we decode the stimulus from each layer by using the population spikes binned at various time resolutions Δt using two types of recurrent neural networks (see decoding analysis methods). The association between the true stimulus s and decoded stimulus $\overset{ˆ}{s}$ is estimated using various measures, including the mutual information $I_{m}(s,\overset{ˆ}{s})$. Since the decoded stimulus is a function of the response (i.e. $\overset{ˆ}{s}=f(r)$), the data-processing inequality states that $I_{m}(s,\overset{ˆ}{s})\leq I_{m}(s,r)$. Thus, when we quantify how associated s and $\overset{ˆ}{s}$ are, we are computing a lower bound on the true association between stimulus s and response r. Note that the estimated stimulus $\overset{ˆ}{s}$ from each layer and the network readout z are separate quantities: the former is constructed by binning population spike trains at various Δt’s and feeding them to the decoder while the latter is purely a mechanism by which we train the network, thus increasing the information in the deeper layers before performing the decoding analysis with the $\overset{ˆ}{s}$’s.

For a variety of stimuli, we demonstrate how this information changes in each layer as a function of the network structure and timescale of spike counting Δt. When Δt is equal to the duration of the response window T, the input to our decoder is a vector of spike counts across each neuron. When Δt=1ms (1 ms is the time step of our simulations), the input to the decoder is matrix of 1’s and 0’s indicating when spikes occurred at each time step, across all neurons in the population. Due to the loss in dimensionality of the neural representation implied by network convergence, we hypothesize that a temporal code (high information at small Δt but low information at high Δt) will be especially beneficial in bottlenecks. Conversely, large populations post-synaptic to network divergence should have less to gain from temporal codes, since they have high-dimensional representations even with a count or rate code (high information across all Δt’s).

**Figure 2 ∣:**
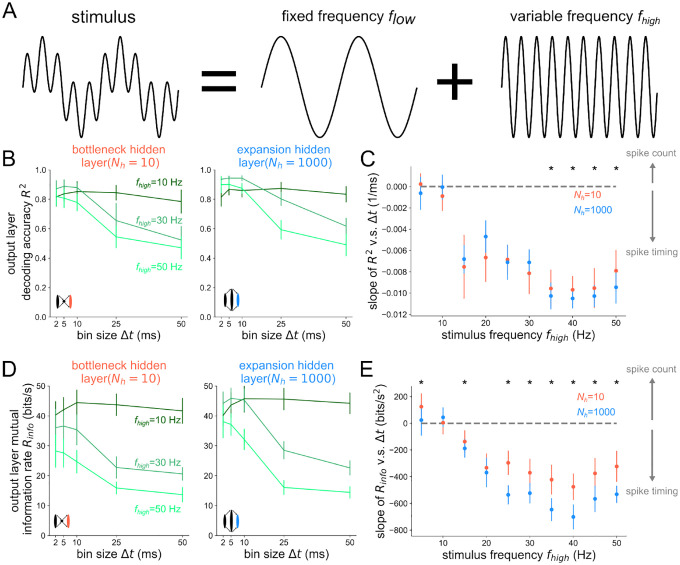
Structural convergence to the output layer promotes timing codes across all stimulus frequencies. (A) Stimuli are sums of sines with fixed frequency component $f_{\text{low}}=4\;\text{Hz}$ and variable component $f_{\text{high}}$ (B) Decoding accuracy based on output layer spikes binned at time resolution Δt. (C) Slope of $R^{2}$ v.s. Δt curves v.s. the high frequency stimulus component $f_{\text{high}}$. Asterisks denote where a one-sided Wilcoxon rank-sum test is significant at p<0.05. (D) Mutual information rate $R_{\text{info}}=I_{m}(s,\overset{ˆ}{s})/T$ based on the output layer spikes binned at time resolution Δt. (E) Slope of $R_{\text{info}}$ v.s. Δt curves v.s. the high frequency stimulus component $f_{\text{high}}$. Asterisks denote where a one-sided rank-sum test is significant at p<0.05.

We first sought to test deterministic stimuli with fixed and well-defined frequency content, opting for sinusoidal stimuli of various frequency. In Fig. 2, the information in the output layer has a steeper decline with growing Δt in the case of the expansion hidden layer structure, especially at higher stimulus frequencies. This is shown for a wide range of stimulus frequencies $f_{\text{high}}$ in Fig. 2C, where the slope of the information curves is plotted as a function of $f_{\text{high}}$. There is a general decrease in the slopes for both bottleneck and expansion networks, owing to progressively better encoding of faster stimuli by spikes binned at higher temporal resolution. Additionally, for all frequencies $f_{\text{high}}\geq 20\;\text{Hz}$ tested, the slope distributions are significantly lower in the expansion hidden layer structure than the bottleneck hidden layer structure. This demonstrates that structural convergence promotes timing codes whereas structural divergence promotes count codes. To ensure that this result did not depend on our specific choices, we tested it for a different decoder and spiking neuron model in Supplementary Section 7.1.1 and found the same result.

**Figure 3 ∣:**
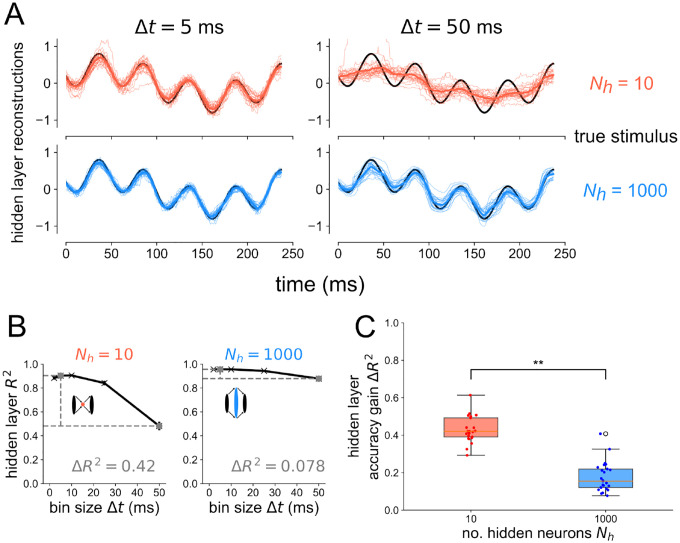
Bottlenecks have more to gain from temporal codes than expansion layers. (A) Example reconstructions from the hidden layer spikes binned at Δt=50ms resolution for $N_{h}=10$ (top) and $N_{h}=1000$ (bottom). (B) Decoding accuracy from the hidden layer spikes as a function of bin size Δt for the bottleneck (left) and expansion (right) network. Gray points denote which bin sizes were used to compute accuracy gain $\Delta R^{2}$. (C) Accuracy gain of the temporal code over count code when reconstructing the stimulus based on spikes from the hidden layer, for bottleneck (red) and expansion (blue) networks. Onesided Wilcoxon rank-sum test $p<6\times 10^{-10}$.

For the same networks tested in Fig. 2, we also performed a decoding analysis on the hidden layer for the case when $f_{\text{low}}=4\;\text{Hz}$ and $f_{\text{high}}=20\;\text{Hz}$ in Fig. 3. As a visual representation of how more precise temporal codes are associated with bottleneck populations of neurons, stimulus reconstructions are shown for $N_{\text{h}}=10$ and $N_{\text{h}}=1000$ in Fig. 3A from spike trains binned at Δt=5ms and Δt=50ms. In the case of an expansion hidden layer $N_{\text{h}}=1000$, there is little difference between Δt=5ms and Δt=50ms; the drop in decoding accuracy when going from a more precise temporal code Δt=5ms to a less precise code Δt=50ms is only $\Delta R^{2}=0.078$ (see right side of Fig. 3B). However, when decoding from the hidden layer of the bottleneck network $N_{\text{h}}=10$, there is a large drop in decoding accuracy when going from a more precise code (Δt=5ms) to a less precise code (Δt=50ms). From Fig. 3A, it is clear that the drop in accuracy comes from the fact that the $N_{\text{h}}=10,\Delta t=50\;\text{ms}$ reconstruction misses the faster 20 Hz frequency component while the other reconstructions do not. By having a higher dimensional representation of the input, the $N_{\text{h}}=1000$ expansion layer can still encode these higher frequency components even with a less precise code, binned over a time window longer than the period of the faster stimulus component. We again tested this result for an alternative spiking model, decoder, and association metric, finding the same general trend in Supplementary Section 7.1.2.

**Figure 4 |:**
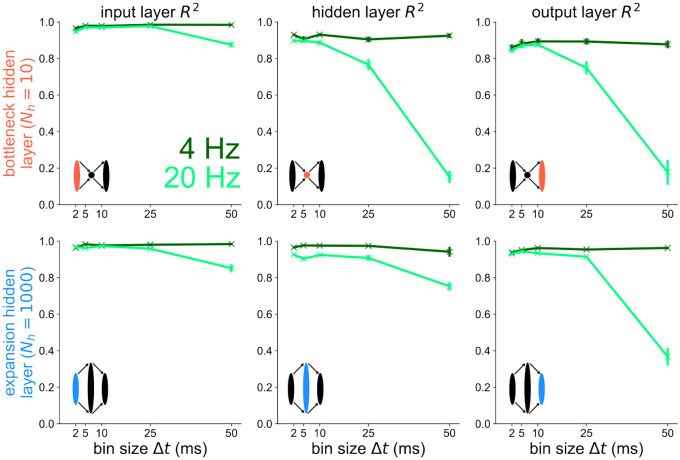
Temporal codes capture high-frequency stimulus components stronger in layers following structural convergence Decoding accuracy versus bin size for each layer of the bottleneck (left) and expansion (right) networks receiving a 4 Hz + 20 Hz sum of sines stimulus. The 4 Hz (dark green) and 20 Hz (light green) components are decoded separately here.

To explicitly show that the higher-frequency component $f_{\text{high}}=20\;\text{Hz}$ contributes to the drop in decoding accuracy for $N_{\text{h}}=10$ at Δt=50ms in Fig. 3, we decode the low frequency component $f_{\text{low}}=4\;\text{Hz}$ separately from the high frequency component $f_{\text{high}}=20\;\text{Hz}$ in Fig. 4 for all layers of the bottleneck and expansion networks. At the input layer (top), there is virtually no difference in the $R^{2}$ v.s. Δt plots between the bottleneck and expansion networks. When decoding from the hidden layer of either the bottleneck or expansion network, the decoding accuracy of the 4 Hz component remains constant for all Δt’s. However, there is a large discrepancy between the bottleneck and expansion networks when decoding the 20 Hz component from the hidden layer: the bottleneck has a steep decrease in decoding accuracy with increasing Δt while the expansion shows a much slower decrease in $R^{2}$ with increasing Δt. Furthermore, going from the hidden layer to the output layer steepens the 20 Hz curve for the expansion network, but leaves the 20 Hz curve for the bottleneck network virtually unchanged. These results support the conclusion that temporal coding of high-frequency components is promoted by structural convergence. Divergence appears to maintain the same coding strategy of the pre-synaptic layer.

**Figure 5 ∣:**
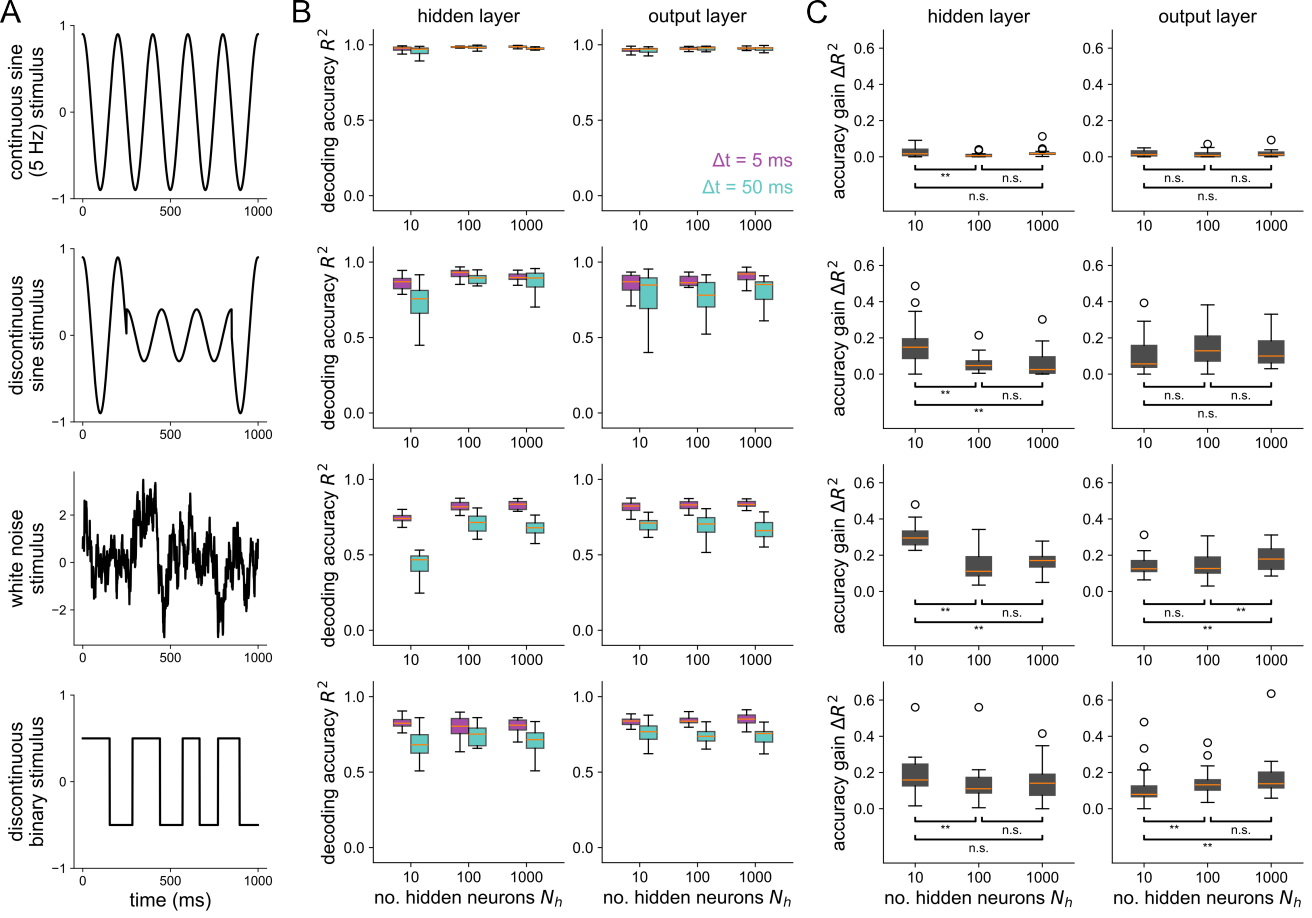
Stimulus-dependence of spike coding as shaped by convergent/divergent structure (A) Each row shows the stimulus used for the corresponding plots on the right. (B) Decoding accuracy v.s. the number of hidden neurons at Δt=5ms and Δt=50ms for the hidden layer (left) and output layer (right). (C) Accuracy gain ($R^{2}$ at Δt=5ms minus $R^{2}$ at Δt=50ms) v.s number of hidden neurons. Asterisks denote where a one-sided Wilcoxon rank sums test is significant at p<0.05.

In the previous results, all stimuli used were sums of 2 sines. In Fig. 5, we show accuracy gains in the hidden and output layer for four different stimuli. For a slow (5 Hz), continuous single sine stimulus (top), there is little to be gained from a more precise temporal code. For the other stimuli shown, which all include some sort of faster time scale or unpredictability, the hidden layer has a higher accuracy gain in a bottleneck network than a uniform $\left(N_{\text{h}}=100\right)$ or expansion $\left(N_{\text{h}}=1000\right)$ network. For the white noise and discontinuous binary stimuli, the output layer has significantly higher accuracy gains in the expansion network than in the networks without structural convergence onto the output layer. Together, these results demonstrate that structural convergence promotes temporal coding in networks responding to stimuli with fast timescales. For slow stimuli without fast jumps, there is little, if anything, to be gained from a temporal code for all network structures tested.

### 2.2 Five-layer network model of the moth visuomotor pathway

Now that a general relationship between optimal coding strategy with spikes and convergent/divergent structure has been established in a simple 3-layer model, we next test our model-based conclusion on this relationship in a specific biological model system of a convergent/divergent neural network found in nature. Specifically, we focus on the visuomotor pathway of the hawkmoth Manduca sexta for its well-defined convergent/divergent architecture along the signal pathway and relative behavioral simplicity during flower tracking. The output of this system consists of only 10 muscles, each acting effectively as a single motor unit or output channel. This compact set of muscles encode the majority of the information about motor output in their spike timing [34].

**Figure 6 |:**
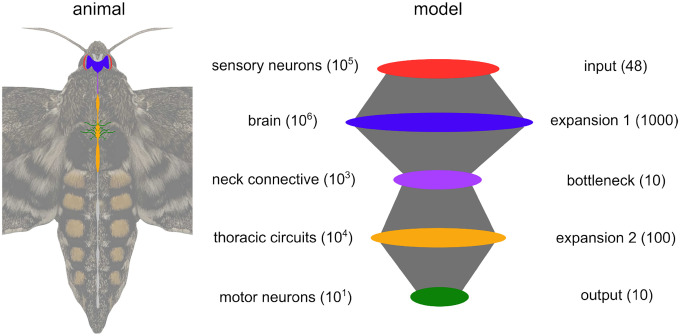
Experimental system and network model Diagram of the central nervous system of the hawkmoth *Manduca sexta* and a schematic of the 5-layered spiking neural network developed here to model its visuomotor pathway. Numbers in parentheses denote the number of neurons in each population for the moth (left) and the model (right).

The output layer provides a nearly complete motor program for behavior allowing for the near perfect (>99%) reconstruction of behavioral output states [53] and between 85% and 95% reconstruction on the continuous 6 degree of freedom (DoF) body forces and torques [54]. The input layer corresponds to the visual system, which we have here simplified as a group of 48 motion-sensitive neurons [33] separated into two subpopulations, each tuned to a direction along a line. Intermediate layers of the moth’s visuomotor pathway include the brain, neck connective, and thoracic circuits. Structurally speaking, each of these populations correspond respectively to an expansion, a bottleneck, and another expansion. For a schematic diagram of the moth’s visuomotor pathway and our corresponding model network, see Fig. 6. The size of each neural population in the model was chosen to preserve the relative order of magnitude of divergence and convergence observed in the moth, within computational capacity.

Our first objective with the 5-layer network was to validate it against previous findings from the motor program of the hawkmoth. In particular, Putney et al [34] performed experiments where hawkmoths were shown a robotic flower oscillating horizontally at 1 Hz, a stimulus that they are naturally inclined to track when foraging. During the flower tracking, the 10 muscles coordinating their flight were recorded with spike timing resolution down to 0.1 ms. The authors found that the majority of the mutual information between the spiking activity of these muscles and the motor output (forces/torques generated during flight) was encoded by spike timing instead of spike count in each unit. Subsequent analysis showed that the spike timing code was 1 ms across all output units [55].

**Figure 7 |:**
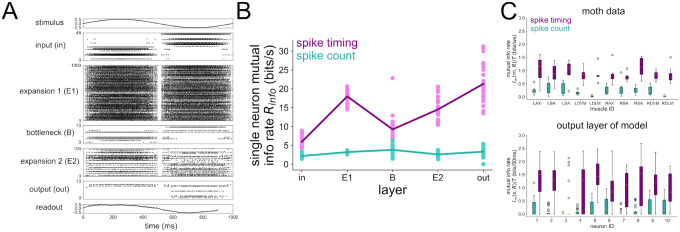
Single-neuron information during 1 Hz stimulus. (A) Raster plot of the 5-layer network model trained to a 1 Hz sinusoidal stimulus. (B) Single neuron information rate in each layer, decomposed into spike count and spike timing contributions. (C) Mutual information in spike count and spike timing from the hawkmoth motor program (top) and the 10 neurons in the output layer of the model (bottom). For the model, mutual info $I_{m}(s,R)$ is taken between stimulus and response; for the moth data, mutual info $I_{m}(m,R)$ is taken between motor output m and response. The single-neuron method depicted in Fig. 1B was used here to compute mutual information, consistent with Ref. [34], which is where the moth data was originally published.

We re-analyzed their data to first confirm this result, shown in Fig. 7C. Next, we trained our 5-layer network model on a 1 Hz stimulus that was used during the experiment and performed the same single-neuron information theoretic analysis for all layers of the model. Since there is no “motor output” from our model, we computed the mutual information between the stimulus and the response, which is analogous to motor output and response in a setting where the stimulus is being physically tracked. The results are shown in Fig. 7. In particular, a large majority of the mutual information in the output layer of our model is encoded by spike timing (bottom of Fig. 7C), just as found from the experimental data (top of Fig. 7C). Furthermore, we show the single-neuron information rate averaged across all neurons within a layer in Fig. 7B. The spike count information is low in all layers compared to the spike timing information. The single-neuron spike timing information starts low in the input layer, rises in the first expansion (E1) layer, falls again in the bottleneck (B), and then rises in the second expansion (E2) and again in the output layer. In the output layer, the spike timing information rate exceeds the spike count information rate by a much larger amount than in the input layer. Note that the information theoretic method used in this analysis is conservative in the sense that contributions from spike timing are only taken once those from spike count have been completely accounted for. Overall, our result lends evidence to the notion that convergent/divergent structure in the hawkmoth visuomotor pathway supports a transformation from the large hidden layer where spike timing is less important to the output layer where spike timing provides an order of magnitude more information than spike count.

**Figure 8 ∣:**
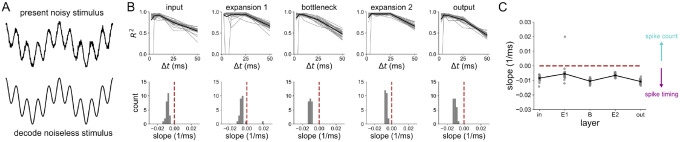
Decoding analysis of a noisy 4 Hz + 20 Hz stimulus (A) Description of the approach: the network receives a sum of sines corrupted by noise, but is trained to encode the noiseless version at the output. The decoding is also done with respect to the noiseless stimulus. (B) Decoding accuracy from spikes binned at resolution Δt, in each layer of the 5-layer model (top) and the distribution of slopes of best line fits to the $R^{2}$ v.s. Δt curves (bottom). (C) Slope distributions versus layer.

Since the 1 Hz sinusoid was decoded very well in all layers and at all time scales (see Supplementary Fig. 16), we sought to investigate what coding strategy was optimal during a more complex and biologically-relevant stimulus. Specifically, we were interested in the idea that the bottleneck may filter the noise in some way. To answer this, we trained the 5-layer network on a noiseless 4 Hz + 20 Hz sum of sines stimulus. Its input was a version of the same stimulus but with white noise added. In each layer, we decoded the noiseless stimulus from population spikes binned at various Δt’s, the results for which are shown in Fig. 8. We found that both expansion layers have a broader range of Δt’s than the smaller layers over which nearly perfect decoding accuracy is achieved. This was quantified by computing the slope of the best line fits to the $R^{2}$ v.s. Δt curves shown on the top of Fig. 8B. The distributions of these slopes for each layer are shown in the bottom of Fig. 8B, and also explicitly against layer in Fig. 8C. A slope of zero means that there is no preference for spike count or spike timing. A negative slope indicates that there is a gain in information with a spike timing strategy over a spike count coding strategy. For the noisy sum of sines used here, all of the slopes (except for one outlier in the E1 layer) were negative. However, the slopes were more negative in the bottleneck and output layer, supporting the conclusion that structural convergence promotes temporal spike coding.

## 3 Discussion

Our 3-layer network results show that bottleneck populations of neurons post-synaptic to a structural convergence have more to gain from precise spike timing codes than expansion layers, so long as the stimulus being encoded has sufficiently fast dynamics. The simple 5-layer network model can reproduce the relative proportion of spike timing information previously deduced at the single neuron level from the motor program of *Manduca sexta*. Notably, the amount by which spike timing information exceeds spike count information at the output layer is vastly higher than that at the input layer. Our model is simplified to capture only the feedforward convergent and divergent architectures of the visuomotor pathway while ignoring other biological mechanisms such as reafference signals, yet replicates the experimental result remarkably well. This suggests that the structure of the hawkmoth visumotor pathway is one that promotes temporal coding at the motor output.

Our work goes beyond previous theoretical studies considering the effects of convergent and divergent structure on information processing [14, 18, 23] by establishing a relationship between this ubiquitous structural motif and the information encoded by spikes at various time resolutions in its constituent neurons. Although the information bottleneck method is useful for understanding artificial neural networks [25–27] and neural data [28], its potential mapping to the discussion of numerical bottlenecks in biological neural networks is unclear. Whereas information bottlenecks are optimal compressions in an abstract sense, numerical bottlenecks of neurons are a structure widely observed in biology that we treat as a starting point and study its consequences for information-processing.

As emphasized above, network convergence and divergence are widespread in several species and brain areas [15–17, 19, 20], but the implication of this structure for spike coding of time-dependent stimuli has not been well characterized. While the importance of spike timing at both sensory input [35–39] and motor output [40–44] is well established, its role in the intermediate processing stages resulting from structural convergence and divergence has been less clear [45, 46, 56, 57]. Our results demonstrate that bottlenecks would benefit greatly from a more temporally-resolved spike code, more so than in expansion layers which have a plethora of neurons to represent a signal with spike counts. This finding is relevant to a variety of systems where cascades of network convergence and divergence are present, including the nervous system of any segmented organism [58].

Whether our findings could be made in alternative learning models is an open question. Although artificial neural network (ANN) models have been used with great success to predict precise spike timing from biologically-relevant stimuli [59], they are unable to make predictions for the role of spike timing in intermediate layers since their units do not have a spiking mechanism. Indeed, this frontier is where past work has delivered mixed results [45, 46, 56] and was important for us to test. Other approaches like the “chronotron” [60] and “tempotron” [61] are single-neuron models that learn to classify inputs with distinct spike timing patterns. However, a training method such as this may bias the coding strategy toward spike timing, which is undesirable when interested in isolating the effect of network structure on coding. For this reason, we chose to train our network in a way that was agnostic to the coding strategy at the output (see Eq. (2)), a notable strength of our approach.

It is possible that our results could depend on the spike generating mechanism in the neurons of interest. For example, the dynamics of resonant-and-fire neurons [62, 63] are selective for stimuli of certain frequencies. This could be especially important in the context of spike timing codes, since patterns of pre-synaptic spikes that resonate with the natural frequency of the post-synaptic neuron will more reliably result in that neuron firing [64]. This is in contrast to leaky-integrate-and-fire (LIF) neurons, which are most likely to spike when the input amplitude is high and the frequency is low. Although we could not test models outside of the LIF family, this particular model in its generalized form has been shown to reproduce a variety of neuronal spiking behaviors [65]. Thus, we expect that the general trends we observe in the two LIF models tested here will extend to other spike-generating mechanisms.

In summary, we found that convergent and divergent structure shapes the way in which populations of neurons encode high-frequency or less predictable dynamic stimulus information with precisely-timed spikes. Bottlenecks resulting from network convergence benefit much greater from precise spike timing than expansion layers coming from network divergence. A simple model recapitulates previous experimental findings at the motor output of the visuomotor pathway of the hawkmoth, supporting our hypothesis that biological neural networks employ the optimal mode of coding with spikes shaped by structural convergence and divergence. While comprehensive experimental data across all layers of the visuomotor pathway is unavailable, our model further makes predictions about unobserved populations and untested stimuli, which could be confirmed experimentally in future studies. Overall, our work establishes a novel structure-function relationship in feedforward neural networks with signal convergence and divergence, elucidating how this structural motif prevalent across neural systems and species determines the optimal coding strategy with spikes.

## 4 Methods

### 4.1 Spiking neuron models

The Python package snnTorch [47] was used to train and run simulations of the spiking neural networks (SNNs) studied here. The spiking neuron model that is used for all primary results is the spike response model or “alpha” neuron. We have also implemented a simple leaky integrate-and-fire neuron model, to verify that the main results are not modeldependent.

The evolution of the alpha neuron is governed by the following difference equations:

(3) $$I_{\text{exc}}[t+1]=\alpha I_{\text{exc}}[t]+I_{\text{in}}[t+1]$$

(4) $$I_{\text{inh}}[t+1]=\beta I_{\text{inh}}[t]-I_{\text{in}}[t+1]$$

(5) $$U[t+1]=\tau_{\alpha }\left(I_{\text{exc}}[t+1]+I_{\text{inh}}[t+1]\right)$$

where α is the decay rate of the excitatory current $I_{\text{exc}}$ and β is the decay rate of the inhibitory current $I_{\text{inh}}$. The term $I_{\text{in}}$ represents external current, which either comes from a stimulus or pre-synaptic spiking. The time constant for the membrane potential U is given by $\tau_{\alpha }=\frac{\text{ln}\alpha }{\text{ln}\beta }-\text{ln}\alpha +1$. To ensure that positive inputs increase the membrane potential, we set α>β.

The leaky integrate-and-fire (LIF) neuron is governed by

(6) $$U[t+1]=\beta U[t]+I_{\text{in}}[t+1]$$

where β is the decay rate of the membrane potential U and $I_{\text{in}}$ is the input current. For both the alpha and LIF neuron models, we set $U[t+1]=U_{\text{reset}}$ whenever the membrane potential reaches threshold $U[t]>U_{\text{thr}}$.

Inspired by motion-sensitive neurons in the fly visual system [66], we designed the input layer of our models to be tuned to various regions of a 2-dimensional plane (the visual scene). For the $i^{\text{th}}$ neuron in the subpopulation of the input layer tuned to quadrant q, the input it receives is

(7) $$I_{\text{in}}^{q,i}=\left\{\begin{array}{ll} \sqrt{s_{x}^{2}+s_{y}^{2}} & \text{if}\left(s_{x}-s^{q,i},s_{y}-s^{q,i}\right)\in \text{quadrant}q\\ 0 & \text{otherwise} \end{array}\right.$$

where q=1,2,3,4 and $i=1,\ldots ,N_{\text{in}}/4$. The stimulus s is a time-dependent vector with two components $s_{x}$ and $s_{y}$ denoting x- and y-positions of an moving object. The total number of neurons in the input layer is $N_{\text{in}}$. We generate a set of random offsets $s^{q,i}\sim 𝒩(0,0.1)$ for each quadrant, independently sampled for each neuron. The purpose of this is to encourage smooth transitions between firing of the 4 subpopulations, which is more biologically-realistic than discrete switching.

### 4.2 Network connectivity

All layers besides the input layer of our feedforward network models solely receive inputs from neurons in pre-synaptic layers. The $i^{\text{th}}$ neuron in the $(k+1{)}^{\text{th}}$ layer other than the input layer receives the input current

(8) $$I_{\text{in}}^{i,k+1}[t]=\sum_{j=1}^{N_{\text{pre}}}\;W_{ij}^{k,k+1}X_{j}^{k}[t]$$

where $N_{\text{pre}}$ is the number of neurons in the layer pre-synaptic to neuron i in layer $k,W^{k,k+1}$ is the synaptic weight matrix from layer k to layer k+1, and $X_{j}^{k}[t]=1$ if pre-synaptic neuron j in layer k spiked at time t and $X_{j}^{k}[t]=0$ otherwise. The entries of $W^{k,k+1}$ that are non-zero with probability p are distributed according to $W_{ij}^{k,k+1}\sim 𝒩\left(0,1/\sqrt{pN_{\text{pre}}}\right)$. Thus, excitatory and inhibitory connections are equally probable in our model, and may both exist for the same pre-synaptic neuron and Dale’s law was disregarded for simplification.

### 4.3 Network training

The synaptic weights between layers of spiking neurons in our networks are optimized with backpropagation-thru-time (BPTT) to minimize the following loss function:

(9) $${ℒ}_{\text{MSE}}(z,s)=\frac{1}{N_{t}}\sum_{t=1}^{N_{t}}\;{\left(z_{t}-s_{t}\right)}^{2}$$

where $N_{t}$ is the total number of time points, $s_{t}$ is the true stimulus at time t, and z is a readout from the output layer of the form

(10) $$z=\gamma z_{\text{time}}+(1-\gamma )z_{\text{count}}$$

where γ=0.5 to equally weigh spike count and timing, $z_{\text{time}}=W_{\text{time}}r_{\text{time}}$ and $z_{\text{count}}=W_{\text{count}}r_{\text{count}}$. The matrices $W_{\text{count}}\in R^{N_{\text{out}}\times d_{s}}$ and $W_{\text{time}}\in R^{N_{\text{out}}\times d_{s}}$ are read-out weights whose entries are initialized randomly from a normal distribution 𝒩(0,0.1). The symbol $d_{s}$ denotes the dimensionality of the stimulus dynamics: either $d_{s}=1$ for the 5-layer network or $d_{s}=2$ for the 3-layer network. The quantities $r_{\text{time}}\in R^{N_{t}\times N_{\text{out}}}$ and $r_{\text{count}}\in R^{N_{t}\times N_{\text{out}}}$ are convolutions of the output layer’s spike trains with two different kernels:

(11) $$r_{\text{time}}=K_{\text{time}}*{\text{P}}_{\text{out}}$$

(12) $$r_{\text{count}}=K_{\text{count}}*{\text{P}}_{\text{out}}$$

where * denotes convolution. The binarized population spikes of the output layer ${\text{P}}_{\text{out}}\in B^{N_{t}\times N_{\text{out}}}$ (where B={0,1}) are convolved with the kernels $K_{\text{time}}$ and $K_{\text{count}}$, which are of the form

(13) $$K(t)=\left\{\begin{array}{ll} \text{exp}\left[-\frac{(t-\Delta t/2{)}^{2}}{\sigma }\right] & \text{if}\;0<t<\Delta t\\ 0 & \text{otherwise} \end{array}\right.$$

where Δt=10ms for $K_{\text{time}}\in R^{10\times N_{\text{out}}}$ and Δt=70ms for $K_{\text{count}}\in R^{70\times N_{\text{out}}}$. The standard deviation is set to $=0.1{\;\text{ms}}^{2}$.

**Figure 9 ∣:**
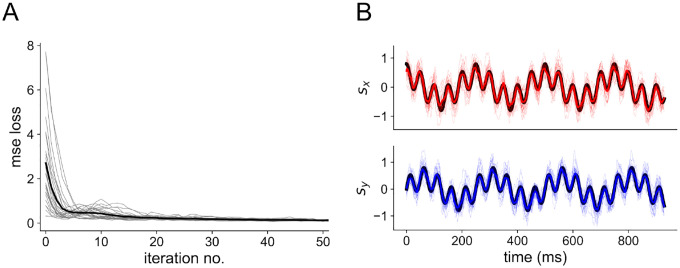
Network training (A) Reduction of MSE loss through training with BPTT. Thin gray traces show individual network seeds, thick black trace shows the average across all 25 seeds. (B) Readout after training 3-layer networks with $N_{\text{in}}=N_{\text{h}}=N_{\text{out}}=100$ to the 4 Hz + 20 Hz sum of sines stimulus. Colored traces are for the readout; the black trace denotes the true stimulus presented to the network. The top shows the x-dimension of the stimulus and the bottom shows the y-dimension.

The read-out weights $W_{\text{time}}$ and $W_{\text{count}}$, as well as the membrane decay rates α and β and synaptic weights (see Section 4) of the spiking neural network, are trained duringback-propagation to minimize the mean-squared error. A plot of the MSE loss over training is shown in Fig. 9, as well as an example of the read-out compared to the stimulus after training.

### 4.4 Decoding analysis

In order to determine how the population responses of layers in our network model relate to stimuli, we trained and tested a decoder [51]. In particular, long short-term memory (LSTM) and gated recurrent unit (GRU) networks were used to predict the stimulus at time t based on the neural response during time [t,t+T] binned at resolution Δt. In other words, the stimulus value at the beginning of the spike train is the value we use the spike train to decode. To further clarify this process, suppose a neural recording of $t_{f}=10$ time steps results in the following spike train:

ρ=[1,0,0,1,1,0,1,0,1,0]

where “0” represents no spike and “1” represents spike. Sliding a rectangular window of width T=8 over this spike train results in

P=[1,0,0,1,1,0,1,0][0,0,1,1,0,1,0,1][0,1,1,0,1,0,1,0]

Each of these are then sub-divided into bins of size Δt. If Δt=T=8, the binned response R becomes a vector of spike counts over the response window T:

R=[4][4][4]

If Δt=4, then the binned response is

R=[2,2][2,2][2,2]

If Δt=2, then the binned response is

R=[1,1,1,1][0,2,1,1][1,1,1,1]

And if Δt=1, then the binned response becomes identical to the original binary spike train P.

The above matrix R has size ($n_{\text{samples}}\times n_{\text{bins}}$) where $n_{\text{samples}}=t_{f}-T+1=10-8+1=3$ and $n_{\text{bins}}=T/\Delta t$. If instead of 1 neuron, we have recordings from $N_{\text{nrn}}$ neurons (as in our population decoding analyses), the same procedure is performed on each neuron’s spike train and their resulting matrices are stacked together to form a tensor of dimension $\left(n_{\text{samples}}\times n_{\text{features}}\times n_{\text{bins}}\right)$ where $n_{\text{features}}=N_{\text{nrn}}$. This tensor is used to decode the stimulus over time. The dimension along which spikes are binned at resolution Δt is treated as a hidden state for the LSTM and GRU decoders, so that decoding depends on specific spike sequences. The stimulus is stored as a matrix S of size $\left(n_{\text{samples}}\times d_{s}\right)$ where $d_{s}$ is the dimension of the stimulus, either 1 or 2 here. The task of decoding is to find a function f that forms an estimate $\overset{ˆ}{S}=f(R)$ of the true stimulus S, minimizing the error $\sum_{i,j}\;{\left({\overset{ˆ}{s}}_{ij}-s_{ij}\right)}^{2}$. In our analysis, the control parameter Δt is varied to modulate the time resolution with which spikes are counted. When Δt=1, there is no difference between P and R, and the specific timing of every spike is preserved. As Δt is increased, spike timings within the larger window of size T become increasingly blurred. The maximum value Δt=T results in a vector R where each entry is the number of spikes that occurred in the respective time window of duration T. On the other hand, as Δt decreases, the code becomes more dependent on spike timing than spike count.

We used the Python package keras to perform the decoding with the LSTM and GRU networks. Cross-validation was performed by maximizing the validation accuracy using Bayesian optimization [67] to select hyperparameters.

### 4.5 Single-neuron information theoretic analysis

We follow Putney *et al*. [34] for the single neuron mutual information analysis. Briefly, their idea was to compute the mutual information $I_{m}$ between motor output m and single-neuron response R via:

(14) $$I_{m}(m,R)=I_{m}\left(m,R_{c}\right)+\sum_{i=1}^{R_{c,\text{max}}}\;p\left(R_{c}=i\right)I_{m}\left(R_{t},m|R_{c}=i\right)$$

where $R_{c}$ is the spike count, $R_{t}$ is the spike timings, and m is the first two principal components of the motor output (forces/torques generated by the wing muscles during hover feeding). The first term in Eq. (14) is what we label the “spike count” information and the second term is the “spike timing” information in the single-neuron analyses in Section 2.2. In our implementation, $R_{c}\in Z_{\geq 0}^{N_{T}}$ where $Z_{\geq 0}$ denotes the set of non-negative integers and $N_{T}=\left(N_{t}/T\right)$ is the number of non-overlapping response windows of duration T falling within the experiment or simulation of duration $N_{t}$. For the moth experiments, T=50ms is the same as the wingstroke period of the animal, so $N_{T}$ equals the number of wingstrokes in this context. The spike timing matrix $R_{t}\in R^{\left(N_{T}\times R_{c,\text{max}}\right)}$ contains the spike timings within each response window where $R_{c,\text{max}}$ is the maximum number of spikes observed in a single wing stroke. The quantity $p\left(R_{c}=i\right)$ denotes the probability that a spike count of i was observed. The mutual information in spike count $I_{m}\left(m;R_{c}\right)$ and the mutual information in spike timing, conditioned on spike count, $I_{m}\left(R_{t},m|R_{c}=i\right)$ were both estimated numerically using the Kraskov-Stögbauer-Grassberger (KSG) method [68] (see Section 4.6). Since there is no “motor output” for our network model, we performed this analysis by substituting the stimulus s for the motor output m in Eq. (14).

### 4.6 Assocation measures

To quantify the amount of information between stimulus and response, we employ various association measures between the true stimulus S and decoded stimulus $\overset{ˆ}{S}=f(R)$ based on the response R. If we define $I_{m}(X,Y)$ as the mutual information between random variables X and Y, the data-processing inequality states that $I_{m}(S,\overset{ˆ}{S})\leq I_{m}(S,R)$ since $\overset{ˆ}{S}$ can only lose information about R [69]. For large populations of neurons and small Δt’s, the response matrix R becomes very high dimensional, rendering the quantity $I_{m}(S,R)$ difficult to estimate directly [70]. Thus, we instead estimate the quantity $I_{m}(S,\overset{ˆ}{S})$ which forms a lower bound on the true mutual information of interest $I_{m}(S,R)$. This is done via the Kraskov-Stögbauer-Grassberger (KSG) method [68], employed via scikit-learn. For the single-neuron mutual information calculations, we used the Julia package CausalityTools.j1. In addition to mutual information, which is a nonlinear measure of association between variables, we also show results with the coefficient of determination $R^{2}$ which is a linear association measure.

## Supplementary Material

1

## Figures and Tables

**Table 1 ∣:** **Parameter values for the neurons in the alpha neuron models.** The symbol 𝒰(A,B) denotes the uniform distribution between A and B.

Alpha neuron parameter initializations		
Parameter name	symbol	value
Excitatory current decay rate	α	𝒰(0.7,0.9)
Inhibitory current decay rate	β	α−0.1
Reset membrane potential	$U_{\text{reset}}$	0
Threshold membrane potential	$U_{\text{thr}}$	𝒰(0,0.5)

**Table 2 ∣:** **Parameter values for the neurons in the LIF neuron models.** The symbol 𝒰(A,B) denotes the uniform distribution between A and B.

LIF neuron parameter initializations		
Parameter name	symbol	value
Membrane potential decay rate	β	𝒰(0.7,0.9)
Reset membrane potential	$U_{\text{reset}}$	0
Threshold membrane potential	$U_{\text{thr}}$	𝒰(0,1.1)
