# Supplementary material for: Temporal resolution of spike coding in feedforward networks with signal convergence and divergence
Source: bioRxiv. 2024 Oct 28:2024.07.08.602598. Originally published 2024 Jul 11. Preprint. [Version 2] doi: 10.1101/2024.07.08.602598 (PMC11257569; doi:10.1101/2024.07.08.602598)
Supplement: 1 — S1 Fig. Version of fig. 2 but with GRU decoder S2 Fig. Version of fig. 2 but with LIF spiking model S3 Fig. Version of fig. 3 but with mutual information. S4 Fig. Version of fig. 3 but with GRU decoder. S5 Fig. Version of fig. 3 but with GRU decoder and mutual information. S6 Fig. Version of fig. 3 but with LIF neuron model. S7 Fig. Decoding analysis of 5 layer model receiving 1 Hz stimulus S8 Fig. Single-neuron information across layer of the 5-layer model for three values of k, the number of nearest neighbors in the KSG method. S9 Fig. Single-neuron information across layer of the 5-layer model for various data set sizes n. S10 Fig. Robustness of the single-neuron information estimates S11 Fig. Interaction info in experimental data v.s. 5-layer model [file NIHPP2024.07.08.602598v2-supplement-1.pdf]

bioRxiv preprint doi: https://doi.org/10.1101/2024.07.08.602598; this version posted October 28, 2024. The copyright holder for this preprint (which was not certified by peer review) is the author/funder, who has granted bioRxiv a license to display the preprint in perpetuity. It is made available under aCC-BY 4.0 International license.

Supporting Information

Generalizability of the three-layer results across spike generators and decoders

Output layer result robustness

This section contains results verifying that our result in fig. 2 is robust to a different spiking (LIF) model and decoder (the gated recurrent unit, GRU).

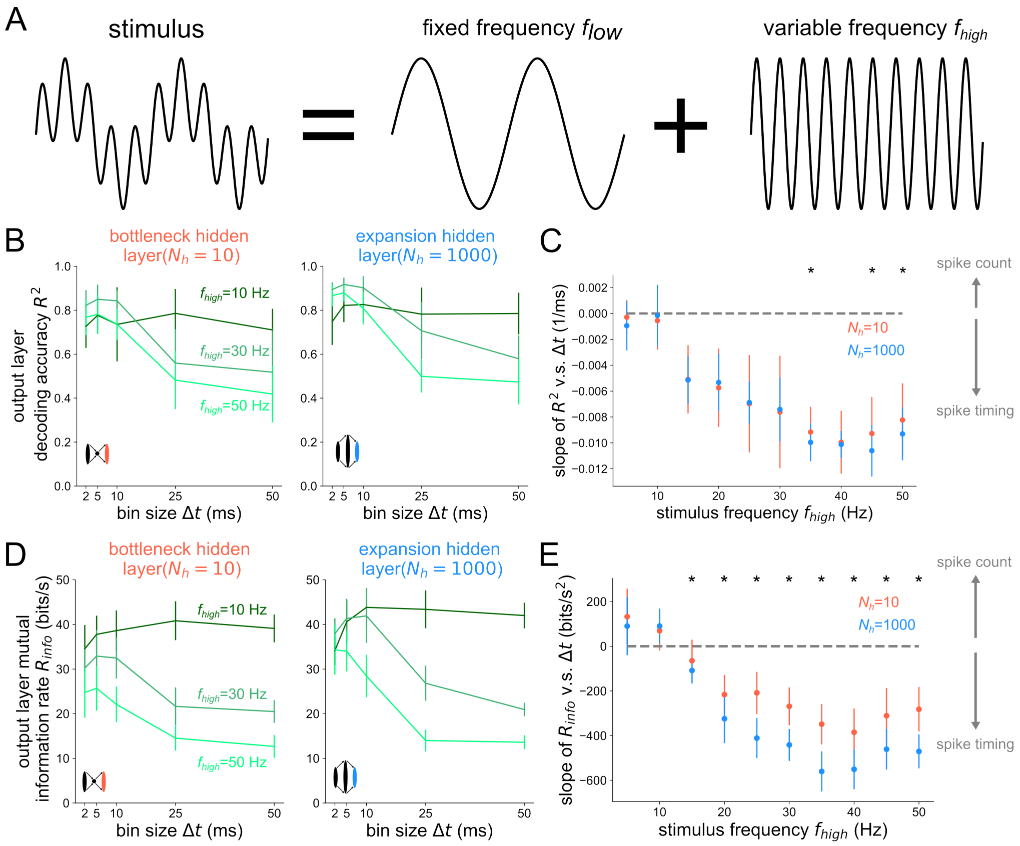

Fig S1. Version of fig. 2 but with GRU decoder

bioRxiv preprint doi: https://doi.org/10.1101/2024.07.08.602598; this version posted October 28, 2024. The copyright holder for this preprint (which was not certified by peer review) is the author/funder, who has granted bioRxiv a license to display the preprint in perpetuity. It is made available under aCC-BY 4.0 International license.

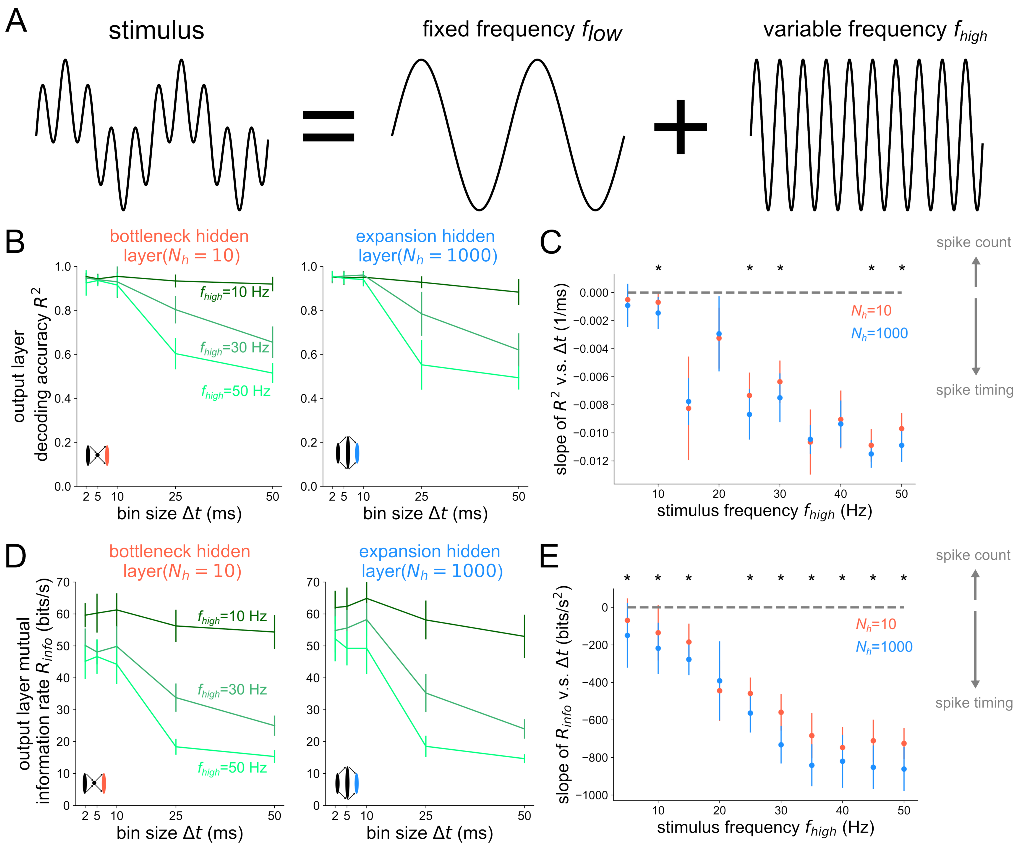

Fig S2. Version of fig. 2 but with LIF spiking model

bioRxiv preprint doi: https://doi.org/10.1101/2024.07.08.602598; this version posted October 28, 2024. The copyright holder for this preprint (which was not certified by peer review) is the author/funder, who has granted bioRxiv a license to display the preprint in perpetuity. It is made available under aCC-BY 4.0 International license.

Hidden layer result robustness

This section contains results that verify the result in fig. 3 is robust to a different spiking model, decoder, and association metric.

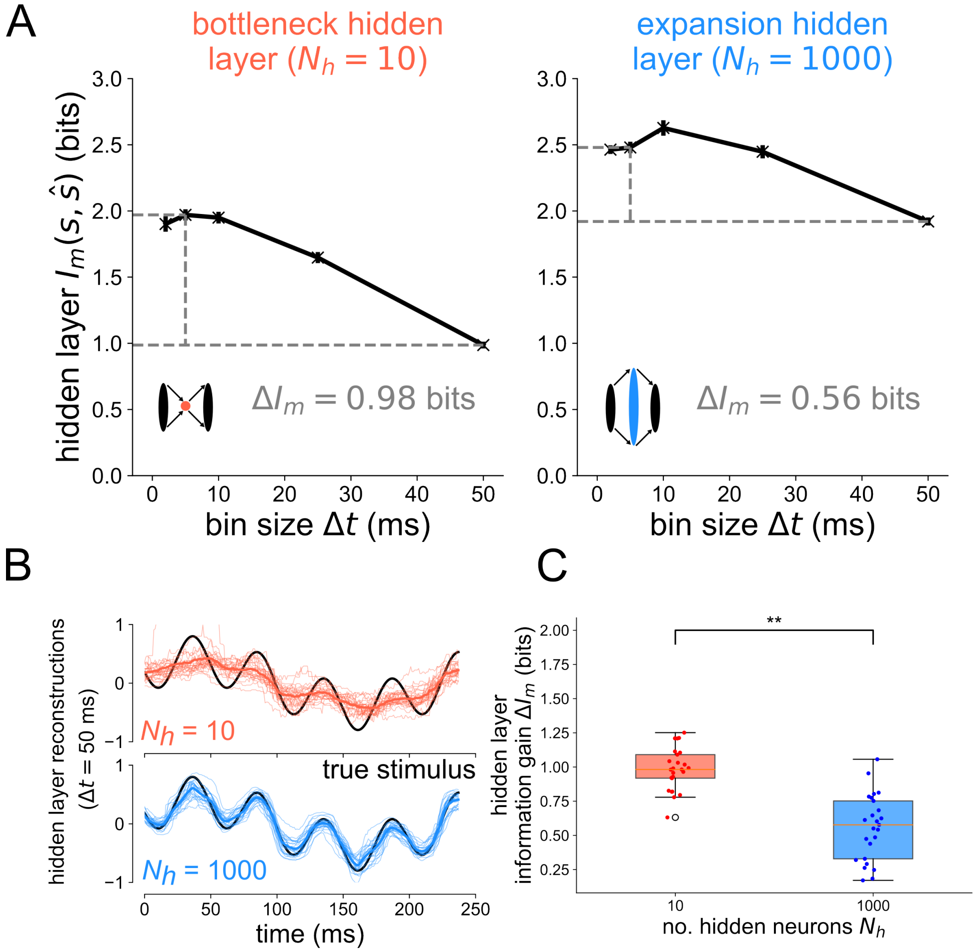

Fig S3. Version of fig. 3 but with mutual information.

bioRxiv preprint doi: https://doi.org/10.1101/2024.07.08.602598; this version posted October 28, 2024. The copyright holder for this preprint (which was not certified by peer review) is the author/funder, who has granted bioRxiv a license to display the preprint in perpetuity. It is made available under aCC-BY 4.0 International license.

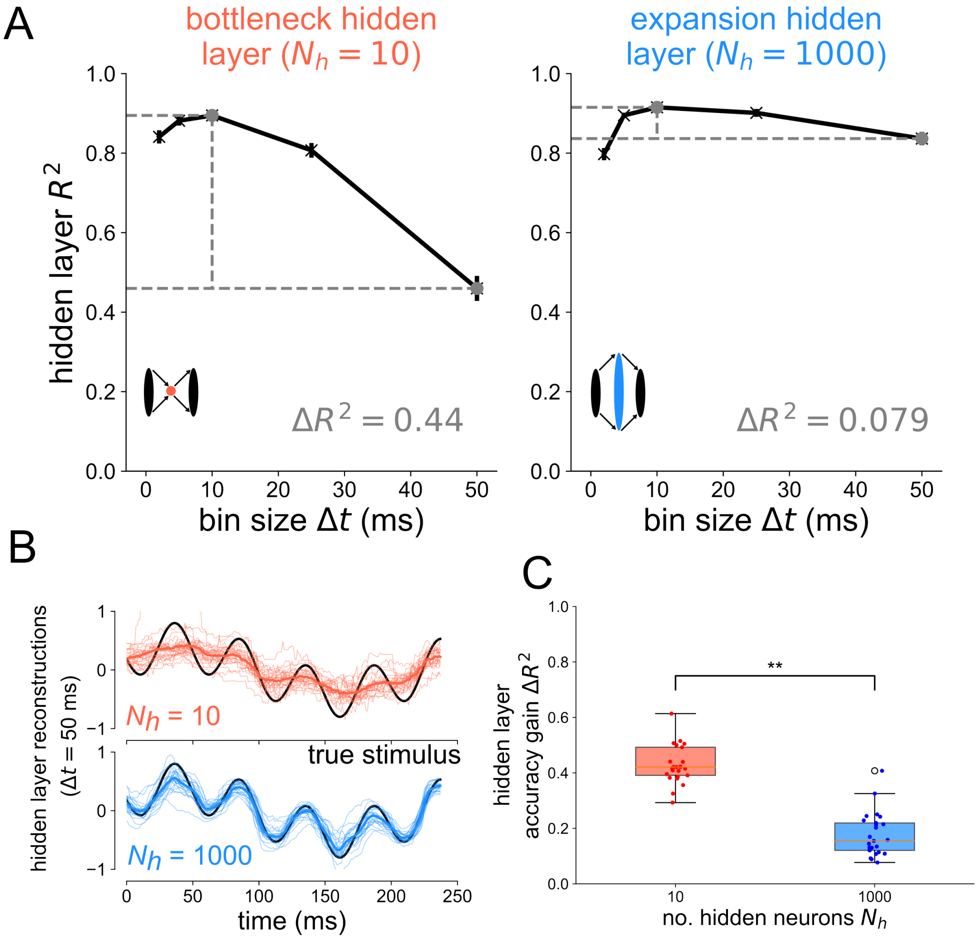

Fig S4. Version of fig. 3 but with GRU decoder.

bioRxiv preprint doi: https://doi.org/10.1101/2024.07.08.602598; this version posted October 28, 2024. The copyright holder for this preprint (which was not certified by peer review) is the author/funder, who has granted bioRxiv a license to display the preprint in perpetuity. It is made available under aCC-BY 4.0 International license.

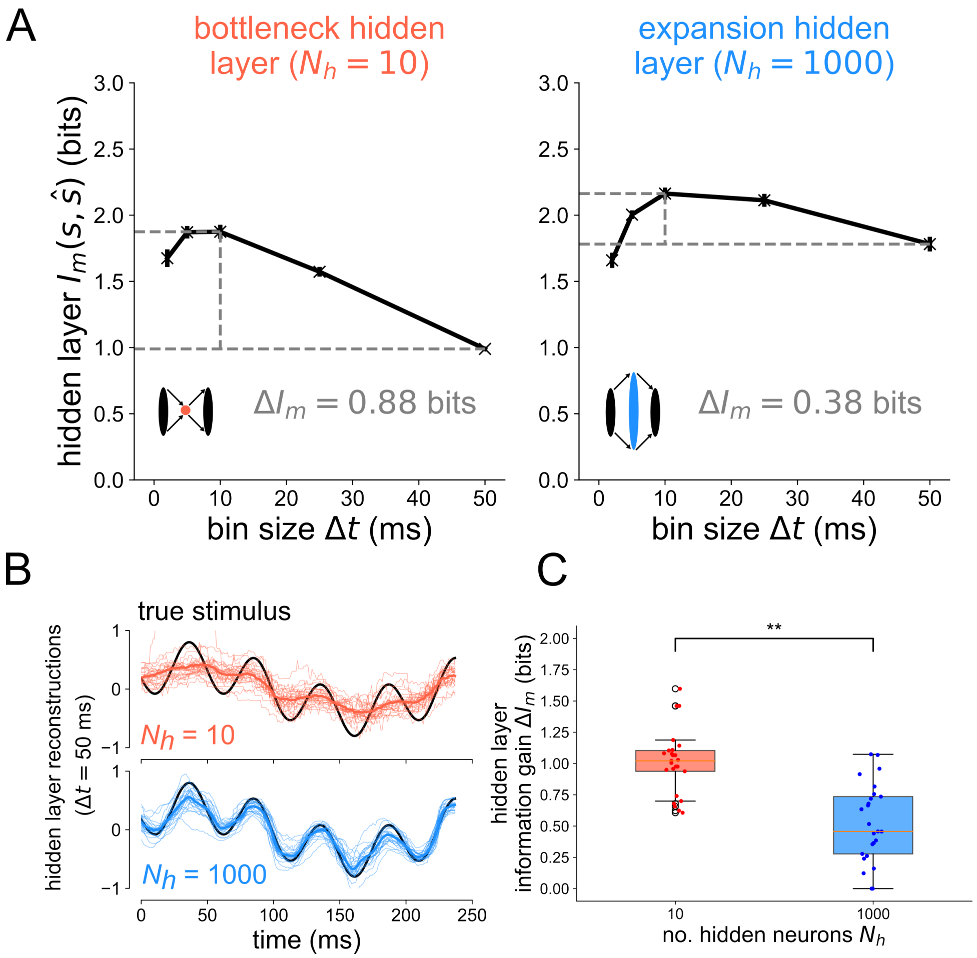

Fig S5. Version of fig. 3 but with GRU decoder and mutual information.

bioRxiv preprint doi: https://doi.org/10.1101/2024.07.08.602598; this version posted October 28, 2024. The copyright holder for this preprint (which was not certified by peer review) is the author/funder, who has granted bioRxiv a license to display the preprint in perpetuity. It is made available under aCC-BY 4.0 International license.

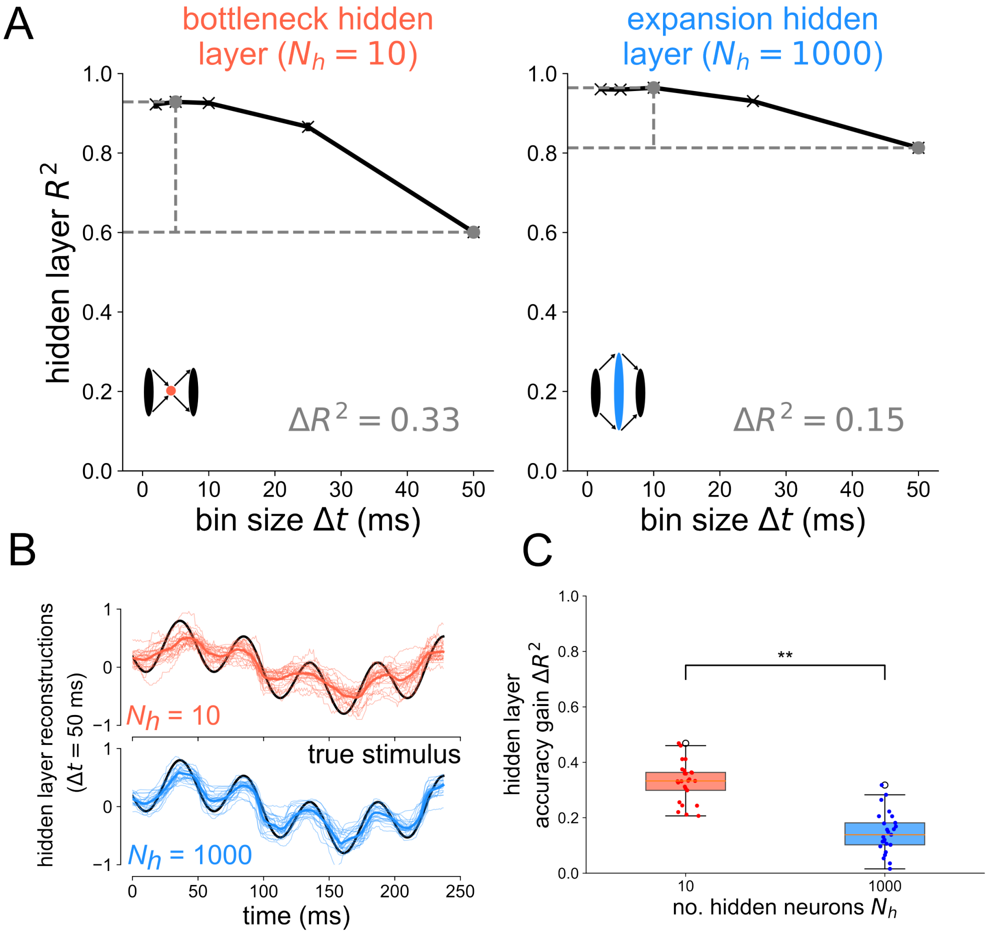

Fig S6. Version of fig. 3 but with LIF neuron model.

bioRxiv preprint doi: https://doi.org/10.1101/2024.07.08.602598; this version posted October 28, 2024. The copyright holder for this preprint (which was not certified by peer review) is the author/funder, who has granted bioRxiv a license to display the preprint in perpetuity. It is made available under aCC-BY 4.0 International license.

Five-layer model 1 Hz stimulus decoding analysis

This section shows the result of the population decoding analysis from the 5-layer network during a 1 Hz sinusoidal stimulus.

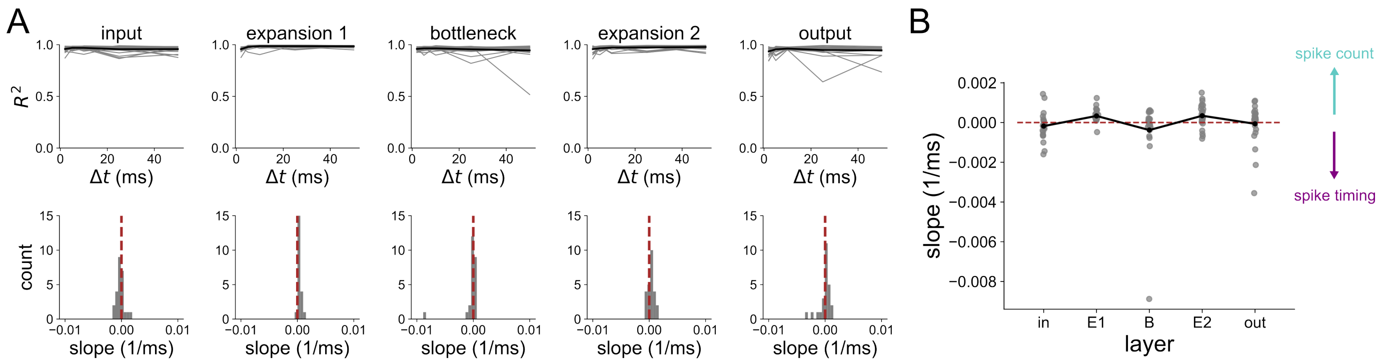

Fig S7. Decoding analysis of 5 layer model receiving 1 Hz stimulus (A) Decoding accuracy R^2 between true stimulus s and estimated stimulus $\hat{s}$ from spikes binned at resolution Δt (top) in each layer of the 5-layer model. Each gray line shows the results from one of 25 network seeds; the black curve is the mean. Slopes of the R^2 v.s. Δt curves in each layer (bottom). Vertical line at slope=0 is plotted for reference. (B) Slope distributions versus layer, gray dots are individual network seeds. The black lines connect the means at each layer.

Single-neuron mutual info robustness

This section contains results verifying that the single-neuron mutual information estimates in 5-layer network are robust to variation in the hyperparameter and dataset. In figure S8 below, we show that the single-neuron mutual information estimates across layer are robust to a range of k, the number of nearest neighbors hyperparameter of the KSG method.

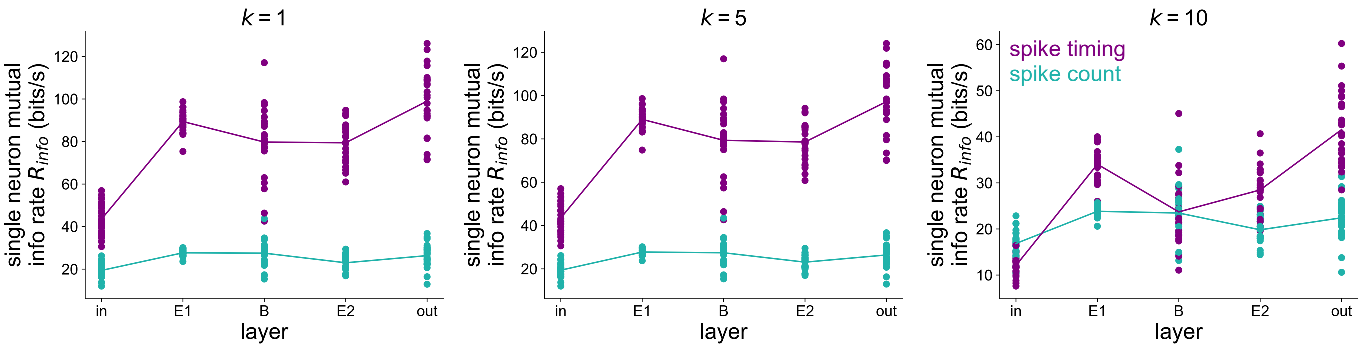

Fig S8. Single-neuron information across layer of the 5-layer model for three values of k, the number of nearest neighbors in the KSG method. Each dot represents the outcome of the single-neuron mutual information analysis for a single 5-layer network seed, pooled across all neurons in the output layer. 25 network seeds used here.

Figure S9 shows how the single-neuron mutual information estimates vary with the number of data points used from the output layer of the hawkmoth visuomotor model trained to a 1 Hz sinusoidal stimulus. The general feature where spike count information is similar to spike timing information in the input layer but much lower than spike timing information in the output layer is preserved across data set sizes.

bioRxiv preprint doi: https://doi.org/10.1101/2024.07.08.602598; this version posted October 28, 2024. The copyright holder for this preprint (which was not certified by peer review) is the author/funder, who has granted bioRxiv a license to display the preprint in perpetuity. It is made available under aCC-BY 4.0 International license.

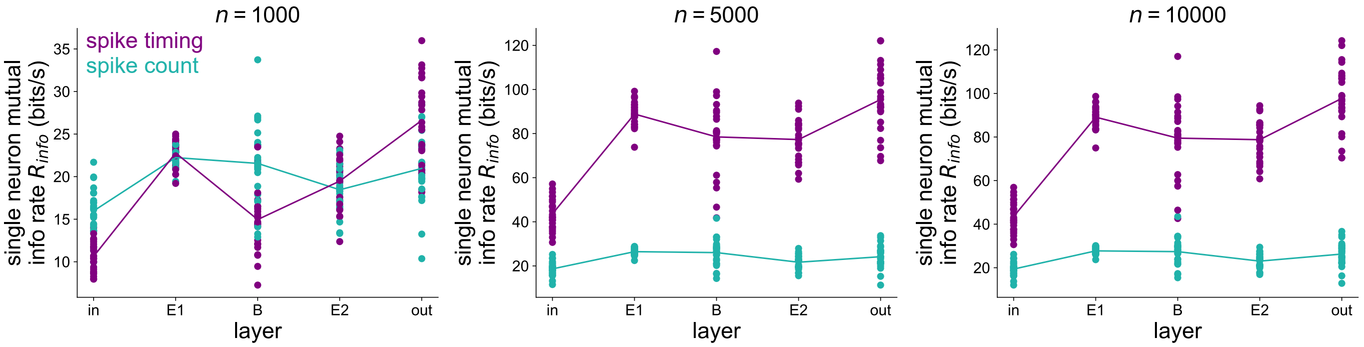

Fig S9. Single-neuron information across layer of the 5-layer model for various data set sizes n. Each dot represents the outcome of the single-neuron mutual information analysis for a single 5-layer network seed, pooled across all neurons in the output layer. 25 network seeds used here.

The single-neuron information analysis of the output layer is shown against k and n in figure S10. Over the ranges of k and n tested, the spike timing information is always significantly higher than the spike count information. In the main results, we use $k = 3$ and $n = 10 \times 10^3$.

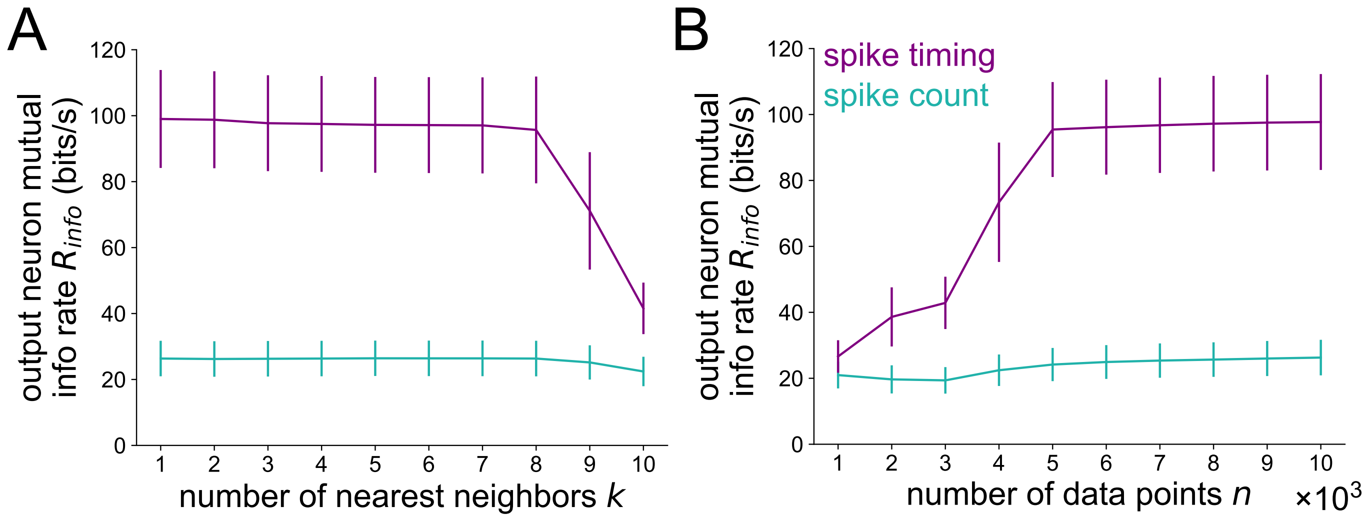

Fig S10. Robustness of the output layer results (A) across number of nearest neighbors k and (B) across data set size n. Error bars represent standard deviations over 25 independent simulations.

Interaction information in hawkmoth model

Another key finding from the previous analysis of Putney et al [25] was that most of the redundancy in the mutual information that moth muscles share with the motor output was in spike timing, not spike count. The authors quantified this through the interaction information between pairs of neurons A and B with motor output m, as defined by

$$II(m; R_A, R_B) = I_m(m; R_A, R_B) - [I_m(m, R_A) + I_m(m; R_B)] \tag{34}$$

where $I_m(m; R_A)$ is the single-neuron mutual information as defined by equation 33 and $I_m(m; R_A, R_B)$ is the joint mutual information that the responses of A and B (R_A and

bioRxiv preprint doi: https://doi.org/10.1101/2024.07.08.602598; this version posted October 28, 2024. The copyright holder for this preprint (which was not certified by peer review) is the author/funder, who has granted bioRxiv a license to display the preprint in perpetuity. It is made available under aCC-BY 4.0 International license.

R_B) share with the motor output m, as defined by

$$I_m(m; R_A, R_B) = I_m(m; R_{A,c}, R_{B,c}) + \sum_{i_A=1}^{R_{A,c_{max}}} \sum_{i_B=1}^{R_{B,c_{max}}} p(i_A, i_B) I_m(m; R_{A,t}, R_{B,t}|(i_A, i_B)) \tag{35}$$

where $R_{A,c}$ and $R_{B,c}$ are the spike counts of neurons A and B, respectively. The maximum spike counts of neurons A and B are $R_{A,c_{max}}$ and $R_{B,c_{max}}$, respectively. The responses $R_{A,t}$ and $R_{B,t}$ are the spike timings of these neurons, which are conditioned on the joint spike count probability distribution $p(i_A, i_B)$. Negative interaction information indicates net redundancy between the pair of neurons. Positive interaction information indicates net synergy. For more details, see ref. [25]. Following Putney et al, we performed this analysis and partitioned the interaction information into spike count and spike timing contributions. We show the results from the original experimental data in the top row of Fig. S11. In the bottom row, we show the result of this analysis performed on the output layer of our 5-layer convergent/divergent network model trained to 1 Hz stimulus. As before, since there is no "motor output" for the model, we use the stimulus s instead of the motor output m in equations 34 and 35, assuming that the motor output reconstructs the stimulus position during tracking. In both the moth data and model data, a majority of the pairwise redundancy is in spike timing instead of spike count, as demonstrated by large negative II_{time} and $II_{count} \approx 0$ in Fig. S11.

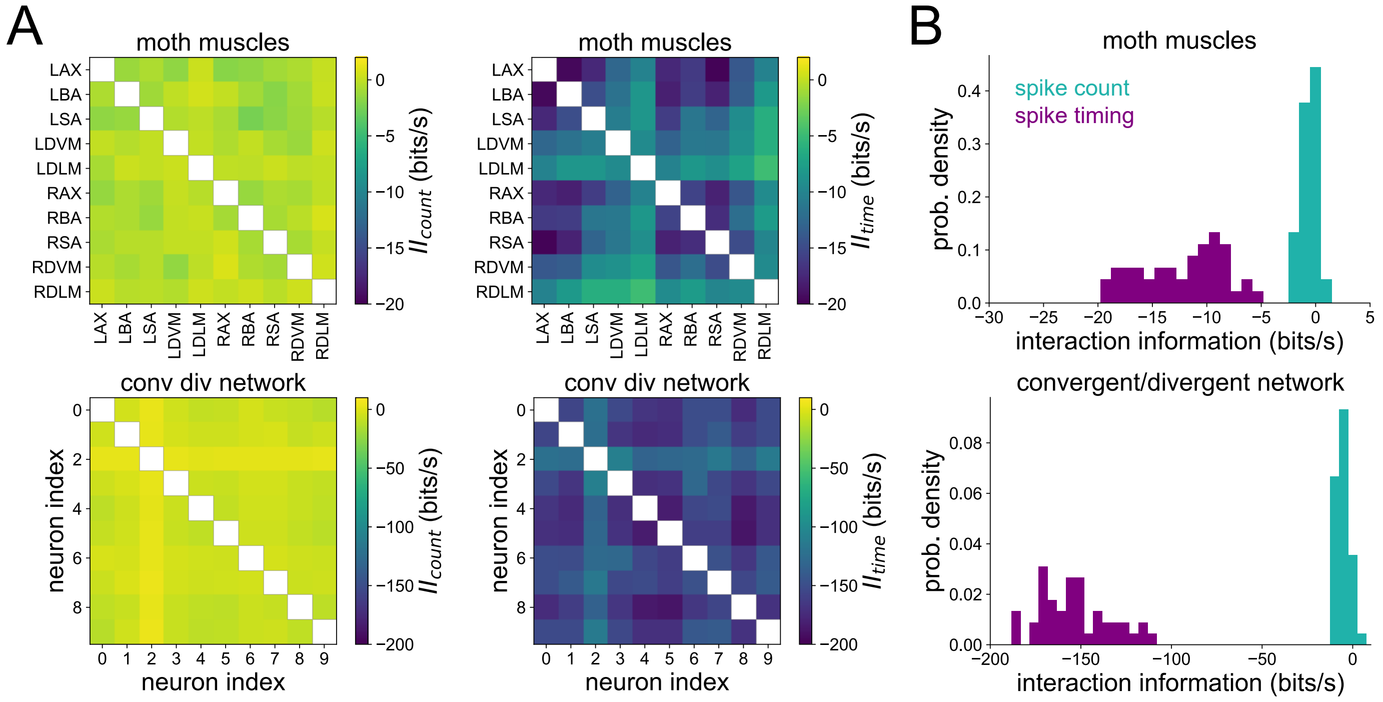

Fig S11. Interaction info in experimental data v.s. 5-layer model (A) Interaction info from spike count (II_{count}) and spike timing (II_{time}) in experimental data (top) and the output layer of our convergent-divergent 5-layer model of the hawkmoth visuomotor pathway (bottom). (B) Interaction info in spike count and timing pooled across all moth muscles (top) and output layer neurons in the model (bottom).
