# Supplementary material for: Temporal resolution of spike coding in feedforward networks with signal convergence and divergence
Source: bioRxiv. 2024 Jul 11:2024.07.08.602598. Preprint. [Version 1] doi: 10.1101/2024.07.08.602598 (PMC11257569; doi:10.1101/2024.07.08.602598)
Supplement: Supplementary file 1 [file NIHPP2024.07.08.602598V1-supplement-1.pdf]

bioRxiv preprint doi: https://doi.org/10.1101/2024.07.08.602598; this version posted July 11, 2024. The copyright holder for this preprint (which was not certified by peer review) is the author/funder, who has granted bioRxiv a license to display the preprint in perpetuity. It is made available under aCC-BY 4.0 International license.

7 Supplementary Information

7.1 Generalizability of the three-layer results across spike generators and decoders

7.1.1 Output layer result robustness

This section contains results that verify our result in Fig. 2 is robust to a different spiking model and decoder (the gated recurrent unit, GRU).

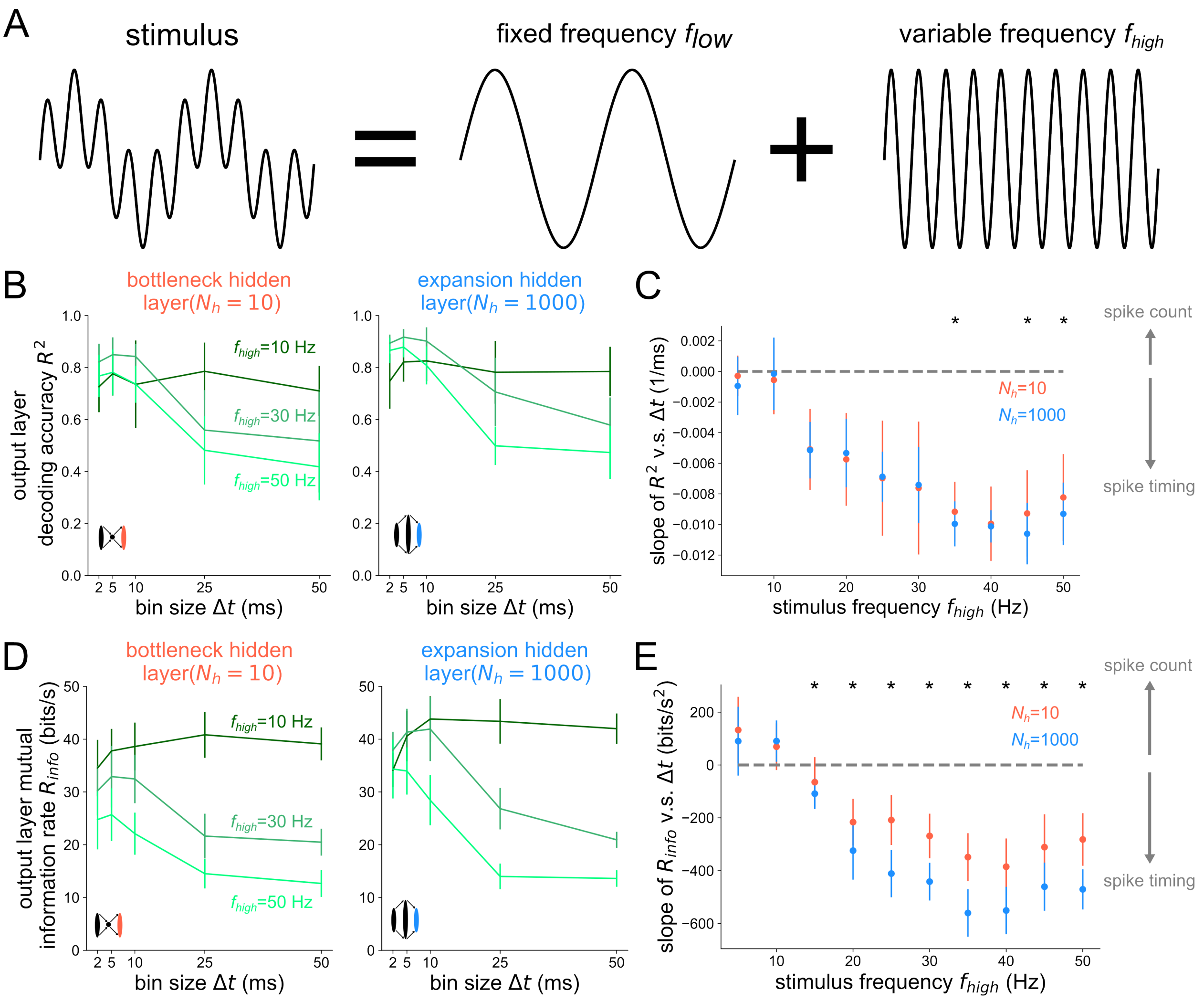

Figure 10 | Version of Fig. 2 but with GRU decoder

bioRxiv preprint doi: https://doi.org/10.1101/2024.07.08.602598; this version posted July 11, 2024. The copyright holder for this preprint (which was not certified by peer review) is the author/funder, who has granted bioRxiv a license to display the preprint in perpetuity. It is made available under aCC-BY 4.0 International license.

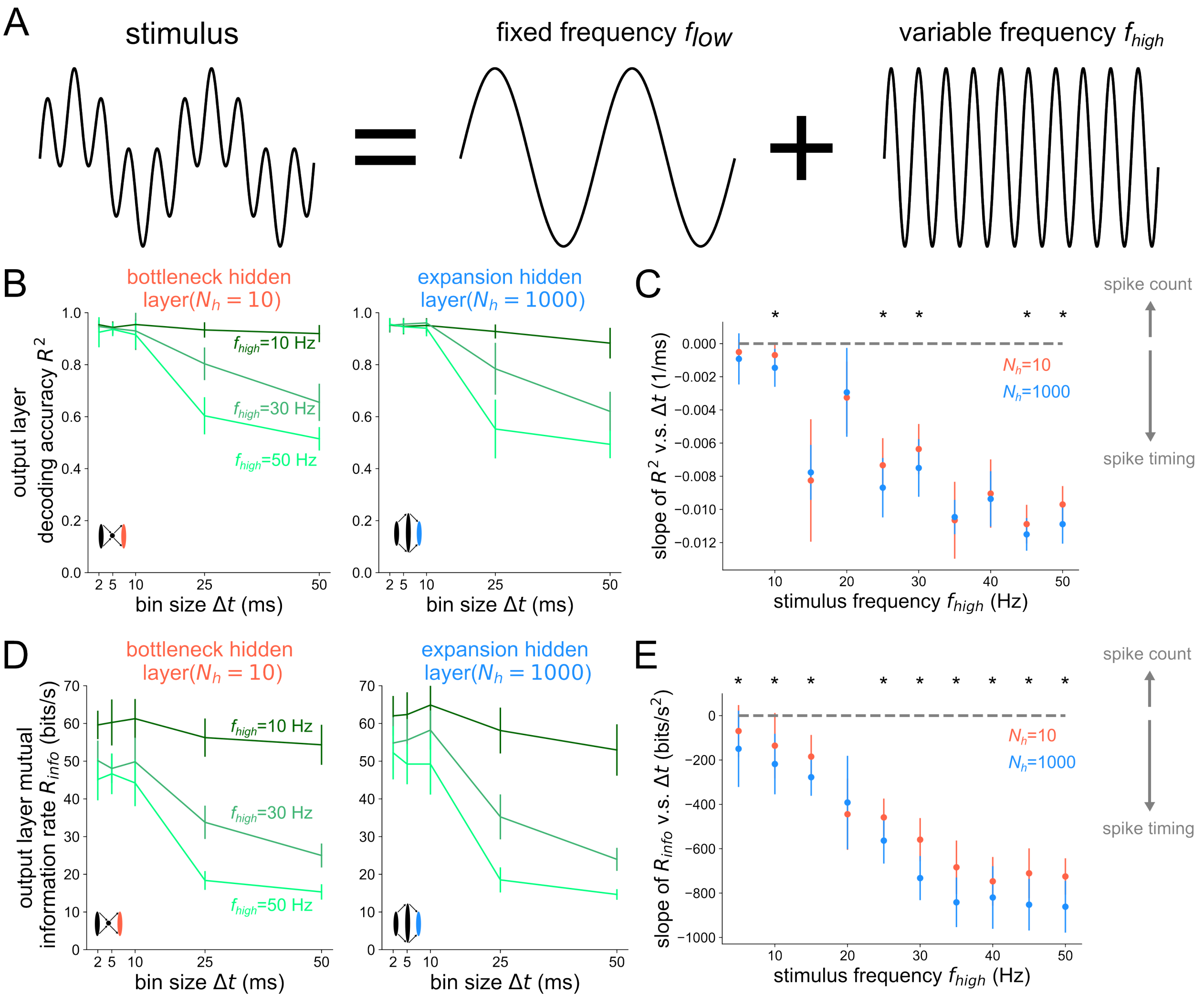

Figure 11 | Version of Fig. 2 but with LIF spiking model

bioRxiv preprint doi: https://doi.org/10.1101/2024.07.08.602598; this version posted July 11, 2024. The copyright holder for this preprint (which was not certified by peer review) is the author/funder, who has granted bioRxiv a license to display the preprint in perpetuity. It is made available under aCC-BY 4.0 International license.

7.1.2 Hidden layer result robustness

This section contains results that verify the result in Fig. 3 is robust to a different spiking model, decoder, and association metric.

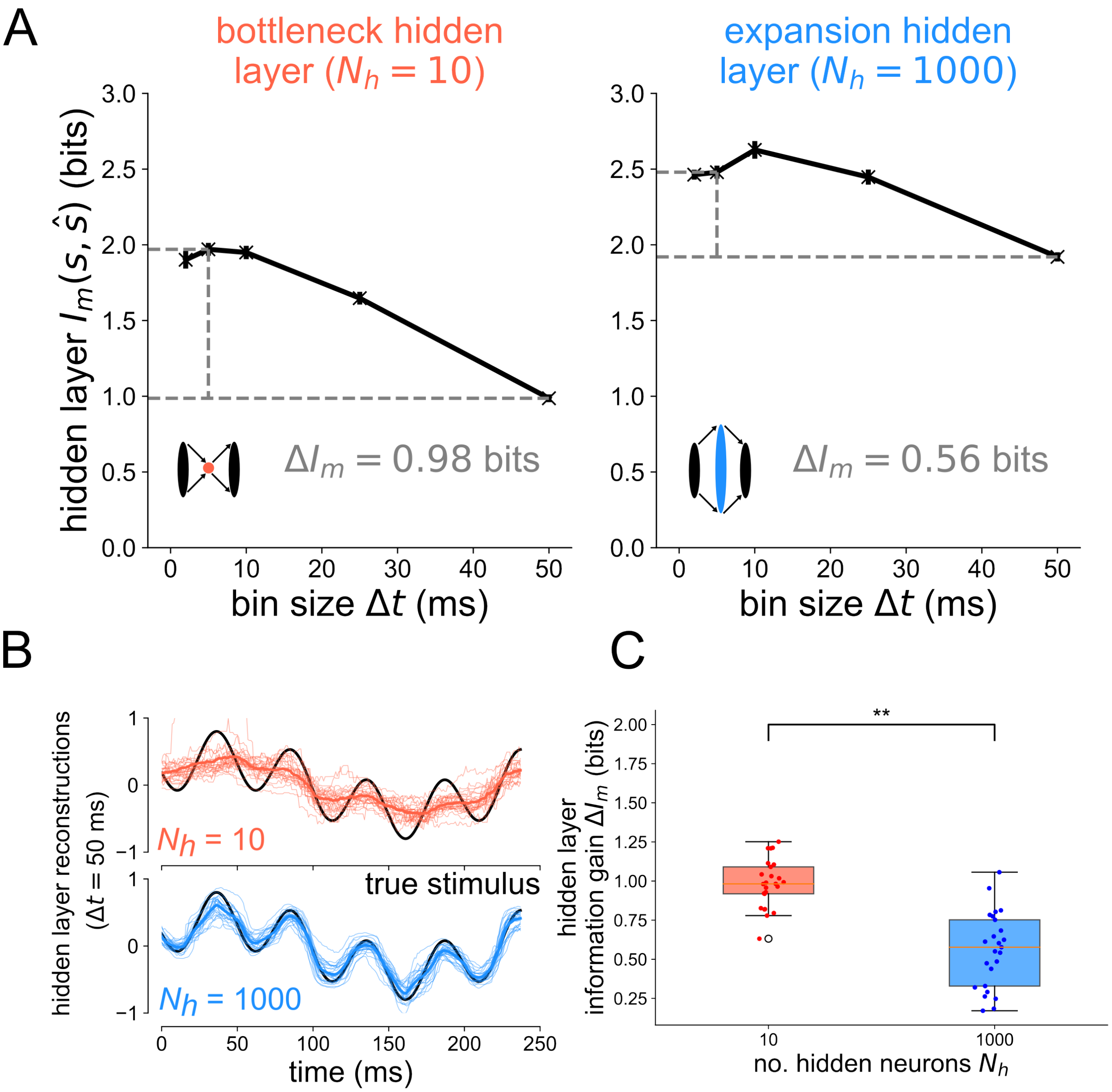

Figure 12 | Version of Fig. 3 but with mutual information.

bioRxiv preprint doi: https://doi.org/10.1101/2024.07.08.602598; this version posted July 11, 2024. The copyright holder for this preprint (which was not certified by peer review) is the author/funder, who has granted bioRxiv a license to display the preprint in perpetuity. It is made available under aCC-BY 4.0 International license.

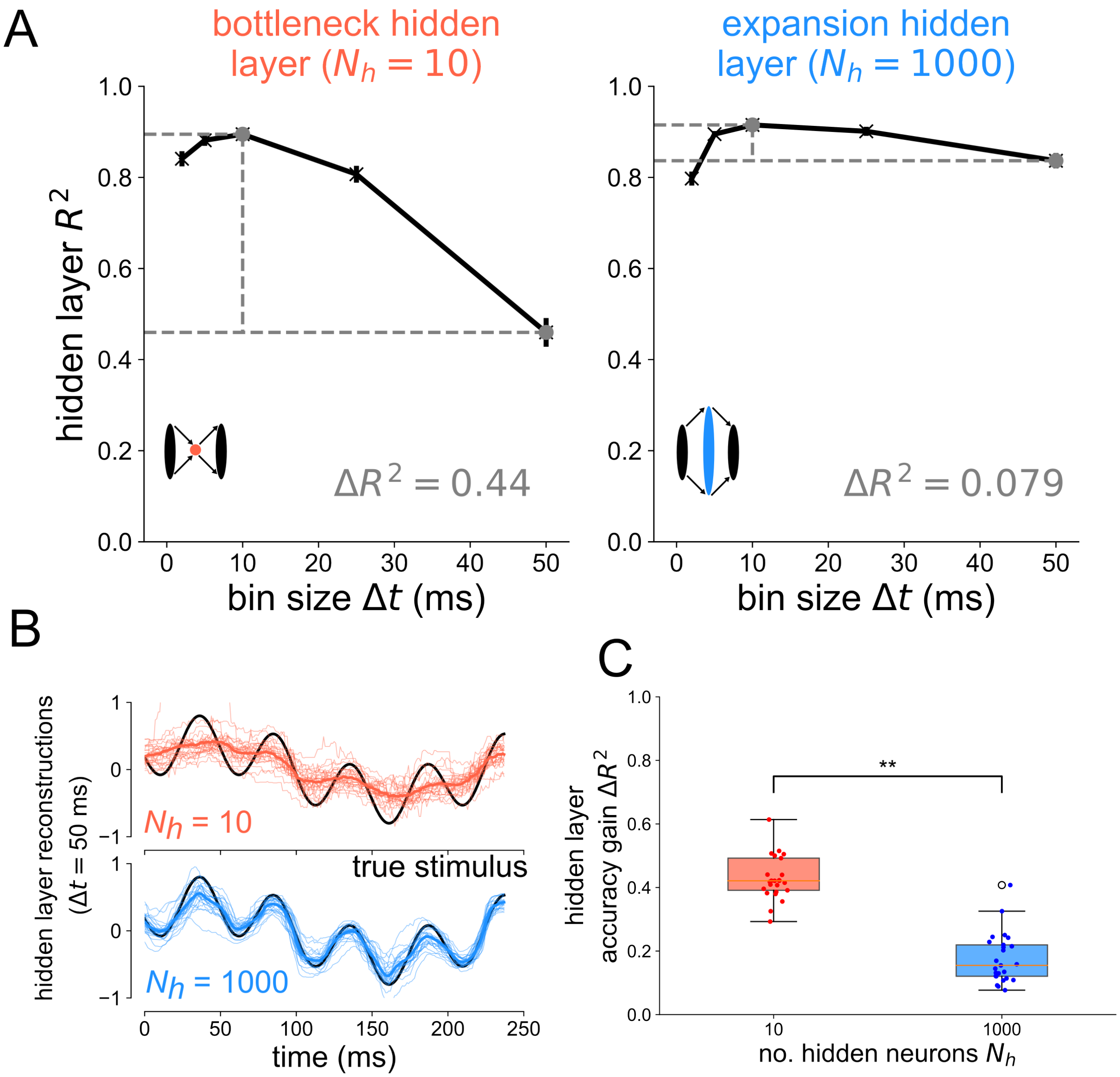

Figure 13 | Version of Fig. 3 but with GRU decoder.

bioRxiv preprint doi: https://doi.org/10.1101/2024.07.08.602598; this version posted July 11, 2024. The copyright holder for this preprint (which was not certified by peer review) is the author/funder, who has granted bioRxiv a license to display the preprint in perpetuity. It is made available under aCC-BY 4.0 International license.

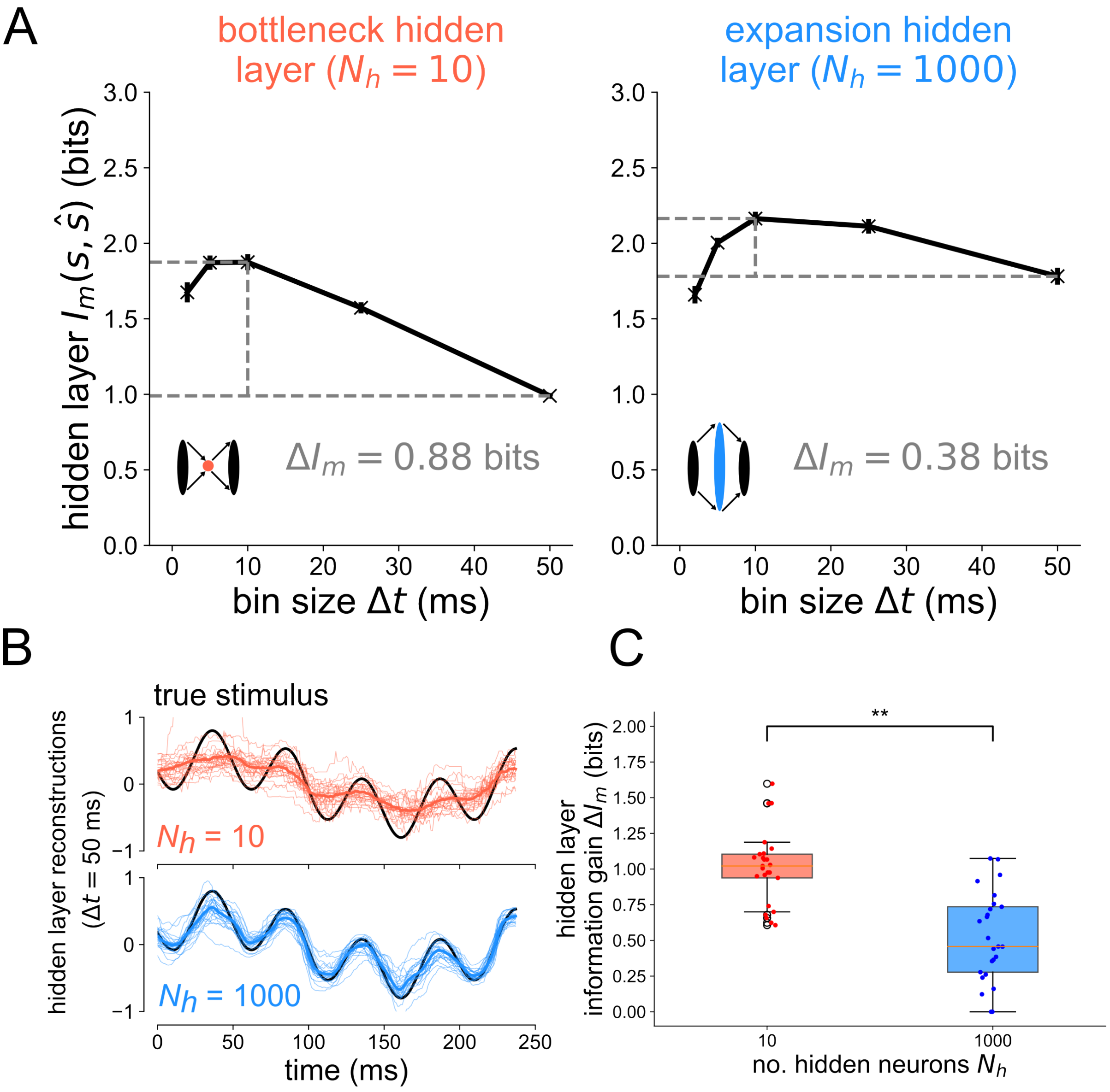

Figure 14 | Version of Fig. 14 but with GRU decoder and mutual information.

bioRxiv preprint doi: https://doi.org/10.1101/2024.07.08.602598; this version posted July 11, 2024. The copyright holder for this preprint (which was not certified by peer review) is the author/funder, who has granted bioRxiv a license to display the preprint in perpetuity. It is made available under aCC-BY 4.0 International license.

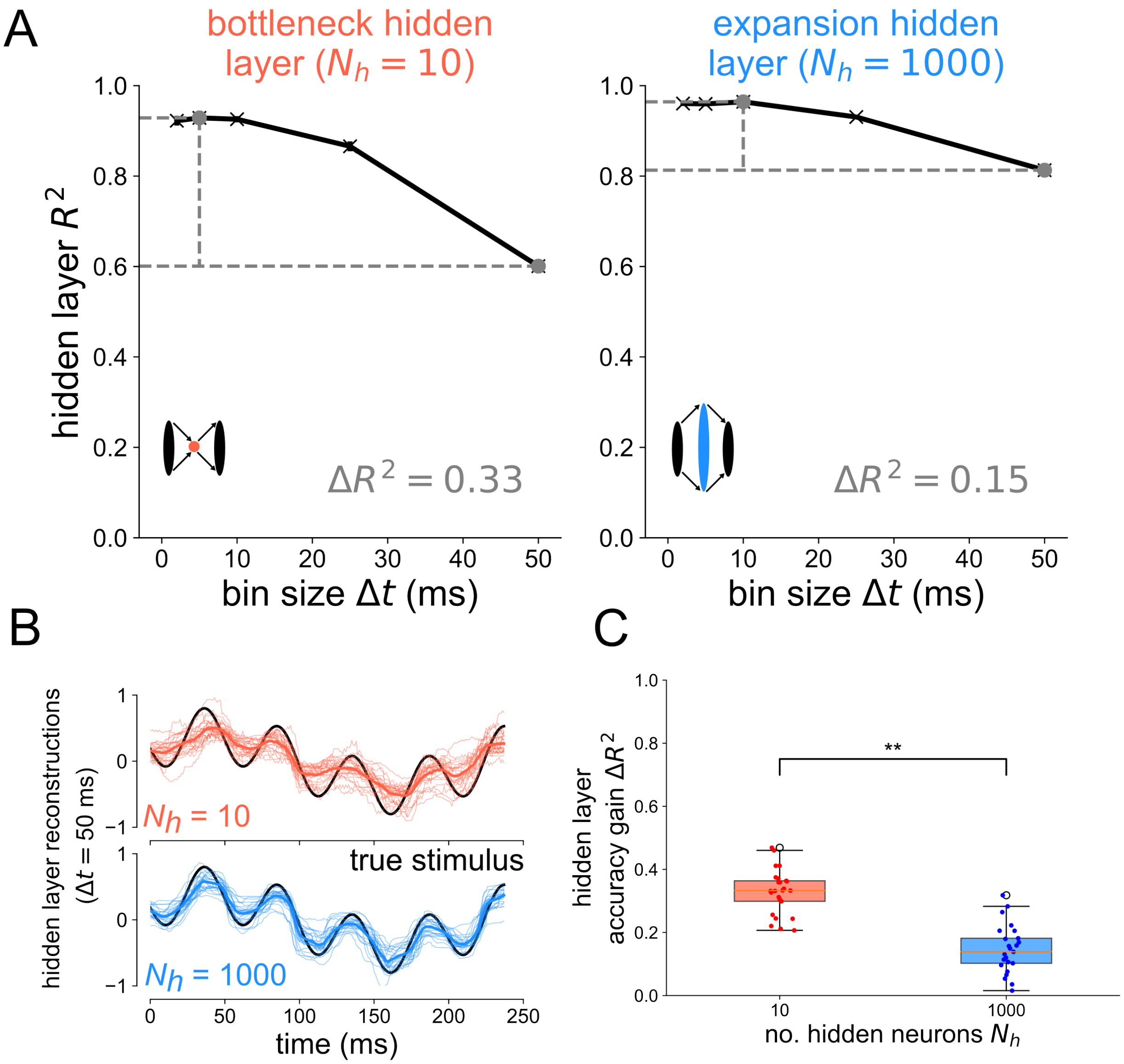

Figure 15 | Version of Fig. 15 but with LIF neuron model.

bioRxiv preprint doi: https://doi.org/10.1101/2024.07.08.602598; this version posted July 11, 2024. The copyright holder for this preprint (which was not certified by peer review) is the author/funder, who has granted bioRxiv a license to display the preprint in perpetuity. It is made available under aCC-BY 4.0 International license.

7.2 Five-layer model 1 Hz stimulus decoding analysis

This section shows the result of the population decoding analysis from the 5-layer network during a 1 Hz sinusoidal stimulus.

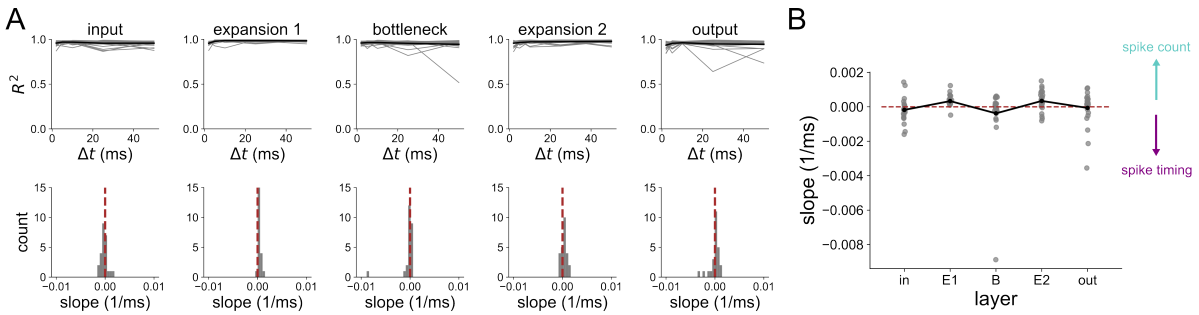

Figure 16 | **Decoding analysis of 5 layer model receiving 1 Hz stimulus** (A) Decoding accuracy R^2 between true stimulus s and estimated stimulus $\hat{s}$ from spikes binned at resolution Δt (top) in each layer of the 5-layer model. Each gray line shows the results from one of 25 network seeds; the black curve is the mean. Slopes of the R^2 v.s. Δt curves in each layer (bottom). Vertical line at slope=0 is plotted for reference. (B) Slope distributions versus layer, gray dots are individual network seeds. The black lines connect the means at each layer.
